# Adaptive compounding speckle-noise-reduction filter for optical coherence tomography images

**DOI:** 10.1117/1.JBO.26.6.065001

**Published:** 2021-06-17

**Authors:** Juan J. Gómez-Valverde, Christoph Sinz, Elisabet A. Rank, Zhe Chen, Andrés Santos, Wolfgang Drexler, María J. Ledesma-Carbayo

**Affiliations:** a Universidad Politécnica de Madrid, ETSI Telecomunicación, Biomedical Image Technologies Laboratory, Madrid, Spain; b Biomedical Research Center in Bioengineering, Biomaterials and Nanomedicine, Madrid, Spain; c Medical University of Vienna, Department of Dermatology, Vienna, Austria; d Medical University of Vienna, Center for Medical Physics and Biomedical Engineering, Vienna, Austria

**Keywords:** optical coherence tomography, denoising, image processing, speckle, wavelets

## Abstract

**Significance:** Speckle noise limits the diagnostic capabilities of optical coherence tomography (OCT) images, causing both a reduction in contrast and a less accurate assessment of the microstructural morphology of the tissue.

**Aim:** We present a speckle-noise reduction method for OCT volumes that exploits the advantages of adaptive-noise wavelet thresholding with a wavelet compounding method applied to several frames acquired from consecutive positions. The method takes advantage of the wavelet representation of the speckle statistics, calculated properly from a homogeneous sample or a region of the noisy volume.

**Approach:** The proposed method was first compared quantitatively with different state-of-the-art approaches by being applied to three different clinical dermatological OCT volumes with three different OCT settings. The method was also applied to a public retinal spectral-domain OCT dataset to demonstrate its applicability to different imaging modalities.

**Results:** The results based on four different metrics demonstrate that the proposed method achieved the best performance among the tested techniques in suppressing noise and preserving structural information.

**Conclusions:** The proposed OCT denoising technique has the potential to adapt to different image OCT settings and noise environments and to improve image quality prior to clinical diagnosis based on visual assessment.

## 1 Introduction

Optical coherence tomography (OCT)^1^ is an optical imaging technique that allows cross-sectional views of *in vivo* tissue in real time with micrometer resolution and at depths of up to two millimeters. OCT is widely used in a variety of biomedical and clinical fields, such as ophthalmology (as a routine noninvasive tool for the diagnosis and monitoring of disease progression)^2^ and cardiology (as a catheter-based imaging system during coronary intervention).^3^ In dermatology, OCT has been used to study a variety of dermatological disorders.^4^^,^^5^ The most significant dermatological applications of OCT are in assessing non-melanoma skin cancers such as basal cell carcinoma (BCC) and in reducing the need for diagnostic biopsies.^6^^–^^8^

Because it uses spatially coherent illumination, OCT images are affected by the speckle phenomenon, which has a dual role as both a source of noise and a carrier of information.^9^^,^^10^ Speckle carrying signal information is the result of the back scattering of the incident photons, whereas speckle noise is caused by the random interference between multiple reflected photons coming from multiple directions. Speckle noise gives a grainy appearance to the OCT images, which degrades the signal-to-noise ratio (SNR) and limits the accuracy of its interpretation. The speckle properties are affected by the scale representation, the optical settings, and the scattering properties of the biological tissue.^11^

Speckle denoising is an active and widespread research field developed during recent years. There are two main approaches: those that modify the image configuration (where the optical setting or the scanning protocol can be adjusted) and those that are based on postprocessing the images via digital algorithms. In the first group, there are three main approaches to the compounding techniques: using the frequency,^12^ using the angle of the incident light source,^13^ and using multiple A-lines collected in a controlled way.^14^ Other studies have proposed methods for combining image processing and modifications to the optical configuration, with the aim of increasing the SNR even further.^15^ To compensate for the effect of possible movement in the case of *in vivo* imaging, two strategies for reducing motion artifacts have been proposed: using a weighted average of individual A-scans^16^ and using a combination of the average of B-scans (from a high-speed CMOS line-scan camera) and a cross correlation of different frames with respect to a fixed reference. One recent report suggests that SNR improvements via pure angular compounding techniques will be limited by optical aberrations.^17^ To overcome these limitations, a recent study proposed the combination of angular compounding with geometric image registration and digital focusing.^18^

The main advantage of digital speckle-reduction techniques is that these can be applied to almost all two-dimensional (2D) and three-dimensional (3D) images acquired by an OCT device without changing the acquisition setup. However, they usually add to the computation requirements and can affect the resolution of the image. These techniques can involve the combined use of several methods such as local averaging over neighboring A-scans of each tomogram,^19^ averaging multiple B-scans,^16^ applying rotation kernel transformations to each tomogram,^20^ image regularization,^21^ complex diffusion filtering,^22^ curvelet transforms,^23^ applying sparse representations or low rank models using patches in the images,^24^^–^^27^ autoencoding based on a neural network that learns from a figure of merit,^28^ digital filtering clusters of pixels with similar optical properties,^29^ and adaptive nonlinear diffusion filtering.^30^ State-of-the-art general-purpose denoising filters such as probability-based non-local-means (PNLM) and block-matching 3D filtering have also been adapted successfully for the removal of noise from OCT images.^31^^–^^33^ Recently, approaches based on deep learning, such as denoising convolutional neural network (DNCNN),^34^ have been proposed for natural image denoising and for speckle noise in OCT,^35^^–^^39^ showing the potential of these techniques in a variety of OCT image types.

Algorithms based on filtering in the wavelet domain^40^ have shown excellent performance in speckle noise removal. One approach used in these methods is to filter the detail wavelet coefficients in multiple subbands to minimize the noise. The calculation of an appropriate threshold and its application can be performed using spatially adaptive soft-thresholding with estimation of the noise in one subband.^41^^,^^42^ However, a recent study shows that speckle noise can have different magnitudes for different wavelet subbands and that estimation of the noise variance at individual scales can improve the characterization of the threshold and can improve the speckle noise removal.^43^ Another successful strategy is the compounding of several frames in combination with digital filtering,^44^ more specifically with frames previously filtered using wavelet denoising.^45^ The use of volumetric data collected from consecutive B-scans has also been exploited by other methods^23^^,^^46^^,^^47^ as an additional source of information for use in speckle reduction.

Here, we present a method for speckle reduction of OCT images that effectively combines an adaptive noise strategy with volumetric wavelet compounding. The method first processes several frames acquired at consecutive locations, using a multiscale noise adaptive wavelet filter, followed by an appropriately weighted computation for the compounding. Following Zaki et al.,^43^ we estimate the noise-variance wavelet representation in a homogeneous scattering sample and use it to filter the noise in all frames. To take advantage of complementary information brought from several acquisitions, we used Mayer et al.’s^45^ compounding approach as a second step, applying a weighting method to select the detail coefficients of each subband before compounding all of the filtered frames. Our method also benefits from the fact that the wavelet representation of the speckle statistics, calculated properly from a homogeneous sample or a region of the noisy volume previously recorded, can be used to characterize the noise pattern of the system. These reference statistics are a valuable asset when differentiating information from speckle noise. In addition, the use of several frames and the wavelet weighted compounding improves speckle removal and enhances the structural details, resulting in a superior performance to that of other state-of-the-art methods.

The rest of the paper is structured in several sections. In Sec. 2, we describe in detail the proposed method, the OCT imaging systems used, the datasets, the performance metrics, and the quantitative evaluation process used in the assessment. In Sec. 3, we report the results and discussion of the parameter assessment and the quantitative evaluation of the algorithm. Finally, Sec. 4 contains the conclusions of the study.

## 2 Materials and Methods

### 2.1 Wavelet-Compounding Adaptive Noise Filter

**Fig. 1:**
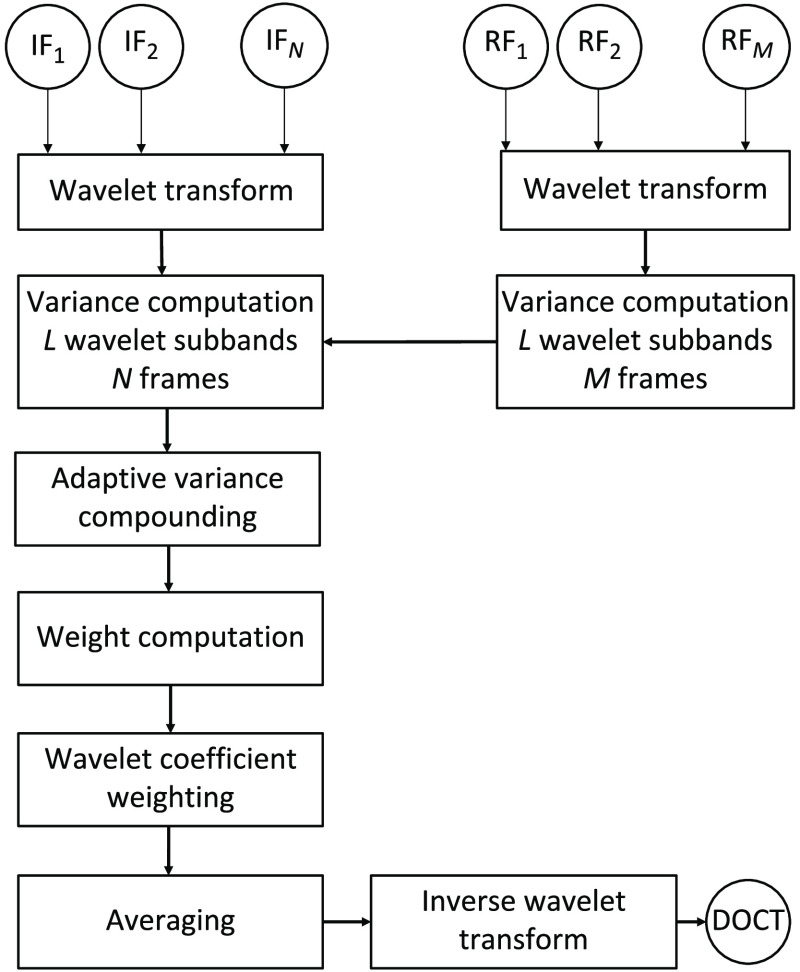
Flow diagram of the wavelet-compounding adaptive noise algorithm. The acquired OCT input frames ${\mathrm{IF}}_{1},{\mathrm{IF}}_{2},\ldots {\mathrm{IF}}_{N}$ represent the N input noisy images for the algorithm. All images and the M reference OCT frames ${\mathrm{RF}}_{1},{\mathrm{RF}}_{2},\ldots {\mathrm{RF}}_{M}$ are wavelet decomposed in L subbands. The variance of all subbands in the wavelet decomposition of the reference stack is computed. Using the subband variances, the weights of the detail coefficients for each subband are calculated based on the compounding of the previous variance computation and applied to each original detail coefficient. All denoised coefficients are then averaged in the wavelet domain before the inverse wavelet transform is calculated to obtain the final denoised OCT image, DOCT.

Figure 1 shows a complete overview of the proposed wavelet-compounding adaptive noise filter (WCAN) method. The processing steps are wavelet transform, variance computation of the input and reference frames, adaptive variance compounding, weight computation, wavelet coefficient weighting, averaging, and inverse wavelet transform. The input to the algorithm is a set of N OCT frames (${\mathrm{IF}}_{1},{\mathrm{IF}}_{2},\ldots {\mathrm{IF}}_{N}$) and a set of M reference OCT frames (${\mathrm{RF}}_{1},{\mathrm{RF}}_{2},\ldots {\mathrm{RF}}_{M}$), with both sets acquired at consecutive positions. The reference frames could come from a homogeneous scattering sample independent of the input frames or from a homogeneous region of the set of input frames. This reference set should be collected as a prior step to the application of the method. The output of the algorithm is a single OCT denoised image. The terms “frame” and “image” from one side and the terms “method,” “filter,” and “algorithm” are used interchangeably in this paper. In the following paragraphs, we explain in detail each step of the method.

#### 2.1.1 OCT image noise

The speckle noise is usually modeled as multiplicative due to the multiple backscattering effects.^48^ In addition to the speckle noise, OCT systems are also affected by noise coming from different sources such as inherent laser intensity noise, photonics shot noise, or thermal noise from electronics. These other types can be assumed to be white Gaussian additive noise.^49^ We define an OCT image $I^{*}$ as

$$I^{*}=N_{s}\cdot I+N_{w},$$ (1)

where I is the noise-free image and $N_{s}$ and $N_{w}$ are the speckle noise and the Gaussian white noise, respectively. We can neglect the additive white noise because it is significantly small compared with the multiplicative noise.^50^ Thus, we model the OCT image as

$$I^{*}\approx N_{s}\cdot I.$$ (2)

To convert the multiplicative noise into additive noise, we consider that all of the OCT images are logarithmic transformed following the approach in similar studies.^50^^–^^54^ This operation allows us to assume a near additive noise model after the logarithmic transformation. We assume also that speckle noise present in frames recorded at different consecutive positions is uncorrelated.^45^

#### 2.1.2 Wavelet transform

**Fig. 2:**
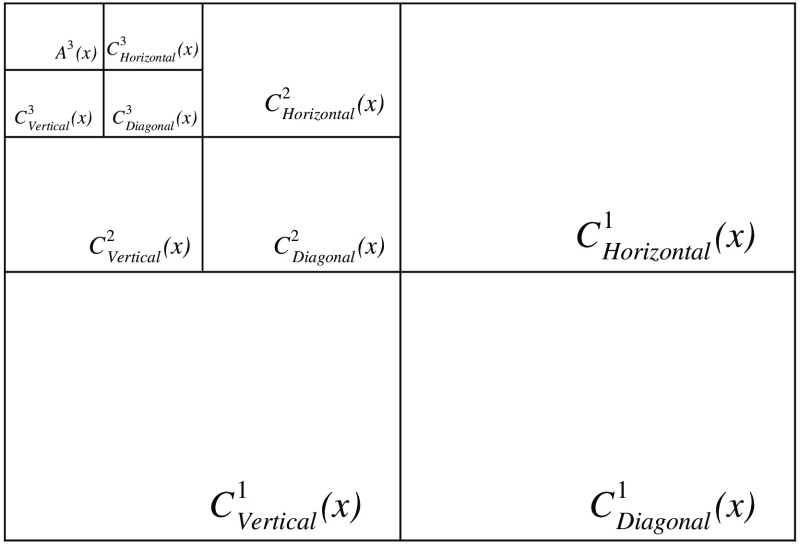
Representation of a 2D wavelet decomposition of an image using three levels.

All of the input images are decomposed by a wavelet transformation with a maximum decomposition level of L. The result of the transformation is a set of approximation coefficients $A_{i}^{l}$ and detail coefficients $C_{i,O}^{l}$, where i is the frame number, l is the decomposition level, and O is the orientation or direction of the detail coefficient (horizontal, vertical, or diagonal). The wavelet transformation is computed using the 2D discrete stationary wavelet transform.^40^ Figure 2 shows a representation of a 2D wavelet decomposition of an image with three levels, where A contains the approximation coefficients and $C_{\text{Horizontal}}$, $C_{\text{Vertical}}$, and $C_{\text{Diagonal}}$ are the detail coefficients of each subband for the horizontal, vertical, and diagonal orientation, respectively.

#### 2.1.3 Variance computation

The next step is the calculation of the variance of the detail coefficients of all of the subbands of the input and reference frames. For the computation of the variance of the detail coefficients of each subband $\sigma_{O}^{l\;2}$, we use the following expressions:

$$\sigma_{O}^{l\;2}=\frac{1}{X-1}\overset{X}{\underset{x=1}{\sum }}{\left|C_{O}^{l}(x)-\mu_{O}^{l}\right|}^{2},$$ (3)

$$\mu_{O}^{l}=\frac{1}{X}\sum_{k=1}^{X}C_{O}^{l}(x),$$ (4)

where l is the subband level (between 1 and L), O is the orientation (horizontal, vertical, or diagonal) of the detail coefficient C, and x is an index ranging from 1 to X (total number of coefficients with orientation O for the subband l). Equation (3) uses $\mu_{O}^{l}$, the mean of all detail coefficients for the orientation O and subband l, calculated using Eq. (4). In the case of the reference frames, we calculate the variance, ${\sigma_{O,R}^{l}}^{2}$, considering all M frames, and therefore, X ranging in this case, from 1 to the number of coefficients by subband and by orientation plus the number of frames M.

#### 2.1.4 Adaptive variance compounding

Then, we calculate the adaptive compounding terms $G_{O,i}^{l}$, which consider an average of the ratio of the variance terms of the initial frames and the reference frames, including also the detail coefficients for the variance of the frame i, using the following expressions:

$$G_{O,i}^{l}(x)=\frac{1}{N}(|C_{O,i}^{l}(x)|\frac{{\sigma_{O,i}^{l}}^{2}}{{\sigma_{O,R}^{l}}^{2}}+\overset{N-1}{\underset{j=1\wedge j\neq i}{\sum }}\frac{{\sigma_{O,i}^{l}}^{2}}{{\sigma_{O,R}^{l}}^{2}}),$$ (5)

where l is the subband, i is the frame number ranging from 1 to N (total number of input frames, see Fig. 1) and O is the orientation of the detail coefficient (horizontal, vertical, or diagonal). The term $C_{O,i}^{l}$ is the detail coefficient of the subband l, orientation O, and frame i. The terms ${\sigma_{O,i}^{l}}^{2}$ and ${\sigma_{O,R}^{l}}^{2}$ are the variance of the detail coefficients from the frame i and from the reference frames respectively, calculated using Eq. (3).

#### 2.1.5 Weight computation

The weights provide estimates of the noise contribution of each subband to every frame in relation to the reference noise and to the remaining frames. The goal is to reduce the detail coefficients in each subband with higher variance compared with the reference values and the remaining frames, assuming that noise will be the main cause of this higher contribution. The term in Eq. 6 is an adaptive variance threshold defined in a similar way to standard wavelet thresholding, but considering the variance of the reference frames in each subband.

$$T_{O,i}^{l}=\frac{{\sigma_{O,R}^{l}}^{2}}{\sqrt{\left|{\sigma_{O,i}^{l}}^{2}-{\sigma_{O,R}^{l}}^{2}\right|}},$$ (6)

$$W_{O,i}^{l}(x)=\{\begin{array}{ll} 1, & |C_{O,i}^{l}(x)|>k\cdot T_{O,i}^{l}\\ 1-G_{O,i}^{l}(x), & \text{otherwise} \end{array}$$ (7)

Equation (7) produces the final weights $W_{O,i}^{l}$ for each subband l, frame i, and orientation O, comparing the value of the detail coefficients $C_{O,i}^{l}$ with the previous threshold value $T_{O,i}^{l}$. The parameter k will then be used to balance the final amount of noise reduction (in Sec. 3.1, we present more detail of the influence and utility of this parameter). The ratio $G_{O,i}^{l}$ from Eq. (5) is used to reduce the weight of the detail coefficients lower than the threshold $T_{O,i}^{l}$.

#### 2.1.6 Wavelet coefficient weighting, averaging, and inverse wavelet transform

The new detail coefficients ${\tilde{C}}_{O,i}^{l}$ of each subband l, orientation O, and initial frame i are computed using the previous weights for all positions x, via the following expression:

$${\overset{˜}{C}}_{O,i}^{l}(x)=C_{O,i}^{l}(x)\cdot W_{O,i}^{l}(x).$$ (8)

The detail $C_{O}^{l}$ and the approximation $A^{l}$ coefficients of the denoised image (see Fig. 2) for each subband l are calculated by averaging the coefficients of the N initial frames considered during the process:

$$C_{O}^{l}(x)=\frac{1}{N}\overset{N}{\underset{i=1}{\sum }}{\overset{˜}{C}}_{O,i}^{l}(x),$$ (9)

$$A^{l}(x)=\frac{1}{N}\overset{N}{\underset{i=1}{\sum }}A_{i}^{l}(x).$$ (10)

Finally, the denoised image is computed by applying the inverse wavelet transform over the averaged coefficients. The algorithm was implemented in Matlab 2018b (MathWorks, Inc., Natick, Massachusetts) on a personal computer (Intel 3.3 GHz CPU, 32 GB memory).

#### 2.1.7 Data and OCT imaging systems

For the quantitative evaluation of the method, we used datasets representing four different environments, as specified in Table 1. Datasets Medical University of Vienna (MUW)-1, MUW-2, and MUW-3 each comprised 18 skin OCT volumes and were acquired via three different OCT image systems. MUW-1 and MUW-2 were custom designs from the Center for Medical Physics and Biomedical Engineering at the MUW. To assess the applicability of the method to a commercial device, the MUW-3 image dataset was acquired via a VivoSight OCT scanner (Michelson Diagnostics, Kent). The A2ASDOCT dataset is a public dataset comprising 17 retinal volumes from the A2ASDOCT study.^59^ It was produced using a Bioptigen Inc (Research Triangle Park, North Carolina) spectral domain OCT imaging system^24^ and involved 17 eyes from 17 subjects with and without nonneovascular age-related macular degeneration (AMD). Each set of images in this dataset comprises five frames of 900×450  pixels from consecutive positions, each of which includes the fovea region. The main technical specifications of the imaging systems are summarized in Table 1. Table 2 gives additional details about the dermatological datasets (MUW-1, MUW-2, and MUW-3) and the retinal dataset (A2ASDOCT).

**Table 1:** Technical specifications of the OCT imaging systems used in the acquisition of the volumes in this study.

Parameter	MUW-1^55^^,^^56^	MUW-2^57^	MUW-3 (VivoSight)	A2ASDOCT (Bioptigen)^58^
System type	Swept-source frequency domain OCT	Spectral domain OCT	Multi-beam swept-source frequency domain OCT	Spectral domain OCT
Laser system	1340 nm	1320 nm	1305 nm Class 1	840 nm
A-line rate	200 kHz	47 kHz	10 kHz	17 kHz
Optical resolution	19.5 μm (air) lateral 4.6 μm (air) axial	15 μm (lateral) 7 μm (axial)	<7.5 μm (tissue) lateral <5 μm (tissue) axial	10 μm (tissue) lateral 4.5 μm (tissue) axial
Field of view	10×10 mm	7×3.5 mm	6×6 mm	6.7×6.7 mm
Imaging depth	1.2 mm	2 mm	Between 1.2 and 2 mm, tissue dependent	2 mm

**Table 2:** Description of the datasets. The volumes of the three dermatological datasets were acquired and evaluated by experts from the Department of Dermatology at the MUW. The identified sample area (if available) is given in the fourth column. The results of the clinical assessments are given in the fifth column.

Dataset	Size (pixels)/number of B-scans	Volume ID	Sample area	Clinical evaluation
MUW-1	1100×512/256	1.1	Hand	Nevus araneus
		1.2	Back	Postsurgical scar
		1.3	Thigh	Normal skin
		1.4	Head	BCC
		1.5	Arm	BCC
MUW-2	1000×680/256	2.1	Palm	Normal skin
		2.2	—	Pityriasis
		2.3	Forearm	Normal skin
		2.4	Head	BCC
		2.5	Arm	Nevus
MUW-3	1558×460/50	3.1	Nose	BCC
		3.2	Chest	BCC
		3.3	Chest	BCC
		3.4	Palm	Normal skin
	1298×460/50	3.5	Fingertip	Normal skin
	1038×460/50	3.6	Back	Nevus
		3.7	Abdomen	Angioma
		3.8	Cheek	Folliculitis
A2ASDOCT	900×450/5	4.1–4.17	Fovea	10 from normal subjects and 7 from AMD subjects

The method requires the use of a reference set (${\mathrm{RF}}_{1},{\mathrm{RF}}_{2},\ldots {\mathrm{RF}}_{M}$ in Fig. 1). The role of this reference is to characterize the speckle noise assuming, as stated by Zaki et al.,^43^ that the variation in these uniform areas is determined by random noise. The calculation of the noise variance is based on Eq. 3 over a region of interest (ROI) selected from the reference frames. We used two strategies to calculate this variance. First, we used a homogeneous scattering phantom made of synthetic clay (Blu-Tak^®^; Bostik, Wauwatosa, Wisconsin). This approach was tested with the MUW-1 OCT image system and the MUW-3 VivoSight device. Using the MUW-1 system, we acquired 512 -scans with dimensions of 512×219  pixels from consecutive positions. Using the VivoSight device, we acquired 120 B-scans with dimensions of 1038×460  pixels from consecutive positions.

**Fig. 3:**
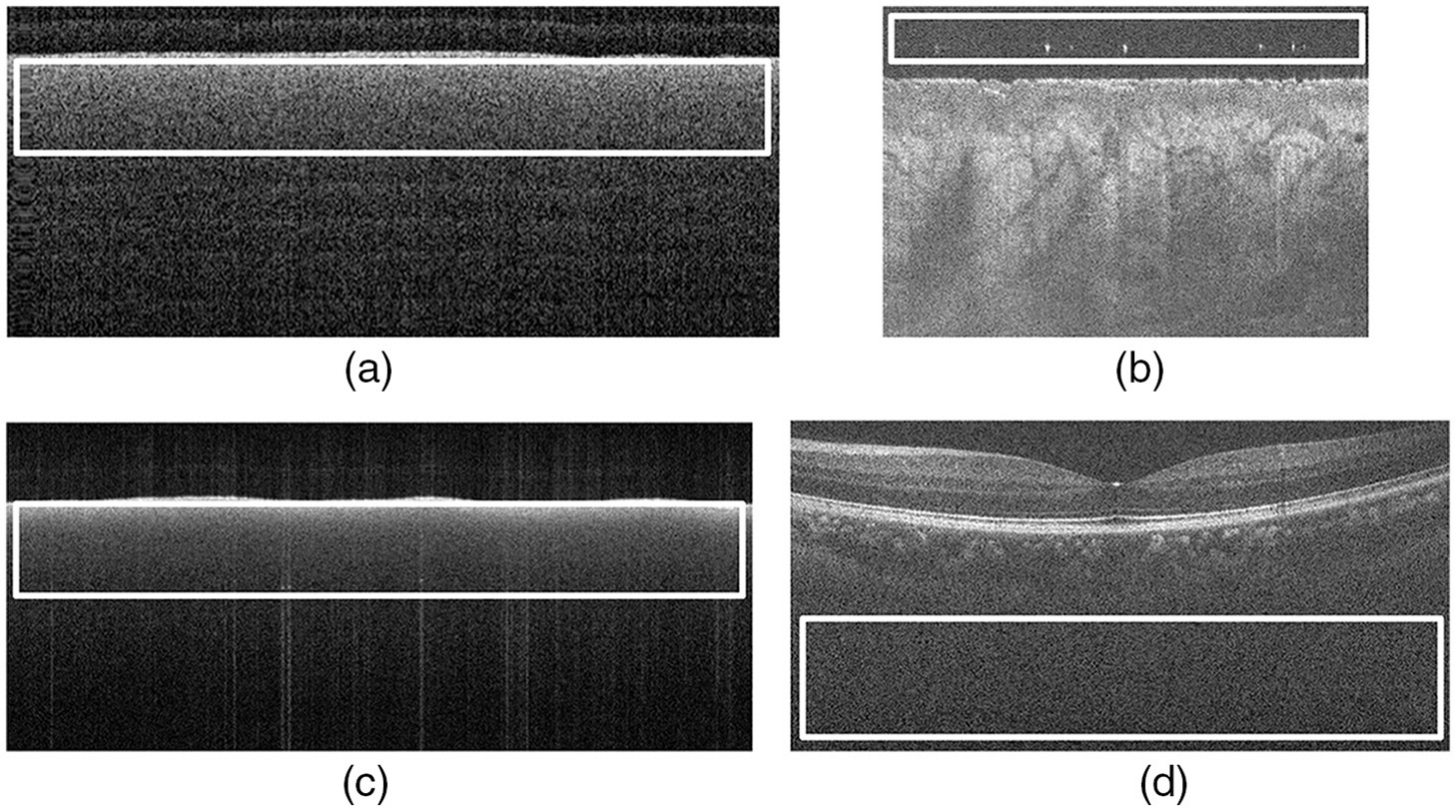
Examples of frames and ROIs (white rectangles) used to compute the noise variance required by the WCAN method. (a) First frame with size 512×219  pixels for the phantom acquired via the MUW-1 image system. (b) First frame with size 1000×680  pixels for Volume 2.1 (palm normal skin, see Table 2) acquired via the MUW-2 image system. (c) First frame with size 1038×460  pixels for the phantom acquired via the VivoSight device. (d) First frame with size 900×450  pixels for the first set in the A2ASDOCT dataset.

In our second strategy, we selected a ROI in the same location (top or bottom) of the frames from both systems and computed the variances of the coefficients of four wavelet detail subbands. This involved selecting a homogeneous noisy ROI and computing the corresponding variance of the detail coefficients, considering all frames in the dataset. We selected the location of the ROI (top or bottom) to have enough data in the all frames of the volume. We used this strategy as an alternative approach for the MUW-2 and A2ASDOCT datasets, partly because a phantom was unavailable and partly to test an alternative approach to noise estimation. Figure 3 shows examples of the acquisition of the phantom and the selection of the ROIs for both strategies, and Table 3 summarizes the reference sets used for each OCT imaging system. We applied the same reference set to all volumes of each dataset (MUW-1, MUW-2, MUW-3, and A2ASDOCT).

**Table 3:** Summary of the reference sets used in the application of the method WCAN for all of the datasets. M indicates the maximum number of frames in the reference set (${\mathrm{RF}}_{1},{\mathrm{RF}}_{2}\ldots {\mathrm{RF}}_{M}$). The ROI Dimensions column refers to the size of the white rectangles shown in Fig. 3.

Dataset	Volume ID	Strategy	M	ROI dimensions	Figure
MUW-1	R.1	ROI phantom	512	63×478	Fig. 3a
MUW-2	R.2	ROI Vol 2.1	256	112×993	Fig. 3b
MUW-3	R.3	ROI phantom	120	120×1009	Fig. 3c
A2ASDOCT	R.4	ROI all frames	85	153×885	Fig. 3d

### 2.2 Quantitative evaluation

We evaluated the efficacy of the algorithm, using common speckle-reduction performance metrics^41^^,^^43^^,^^44^ via a comparison with different state-of-the-art methods. These quantitative metrics were the *SNR*, the contrast-to-noise ratio (*CNR*), and the equivalent number of looks (*ENL*), as expressed below.

$$SNR=10\;\log_{10}(\frac{\mu_{I}^{2}}{\sigma_{I}^{2}}),$$ (11)

$$CNR=\frac{1}{R}\overset{R}{\underset{r=1}{\sum }}\frac{\mu_{r}-\mu_{b}}{\sqrt{\sigma_{r}^{2}+\sigma_{b}^{2}}},$$ (12)

$$ENL=\frac{1}{H}\overset{H}{\underset{h=1}{\sum }}\frac{\mu_{h}^{2}}{\sigma_{h}^{2}}.$$ (13)

*SNR* is defined in Eq. (11), where $\mu_{I}$ indicates the mean of the OCT image and $\sigma_{I}^{2}$ refers to the noise variance. In the definition of *CNR* [Eq. (12)], $\mu_{b}$ and $\sigma_{b}^{2}$ are the mean and variance for a background noise region, respectively, with $\mu_{r}$ and $\sigma_{r}^{2}$ being the mean and variance for all ROIs, respectively, including homogeneous and heterogeneous regions. *ENL* is a measure of the smoothness of a homogeneous ROI. In Eq (13), $\mu_{h}$ and $\sigma_{h}^{2}$ are the mean and variance of all H homogeneous ROIs, respectively. Except for the *SNR* calculations, the remaining parameters were computed from the logarithmic OCT images.

To assess the overall influence of the quantitative metrics, we include two additional figure-of-merits (*FOMs*):^44^

$$FOM_{SUM}=SNR_{Norm}+CNR_{Norm}+ENL_{Norm},$$ (14)

$$FOM_{MIN}=Min(SNR_{Norm},CNR_{Norm},ENL_{Norm}),$$ (15)

where *Norm* refers to the normalization of the metric, that is, the method that performed the best in, for example, the *SNR* criteria, would have an $SNR_{Norm}$ equal to one. Therefore, an $FOM_{SUM}$ of three would indicate that the method performed the best in all image-quality metrics (*SNR*, *CNR*, and *ENL*). The metric $FOM_{MIN}$ is used to assess the robustness of the methods and to detect unbalanced combinations of *SNR*, *CNR*, and *ENL*.

**Fig. 4:**
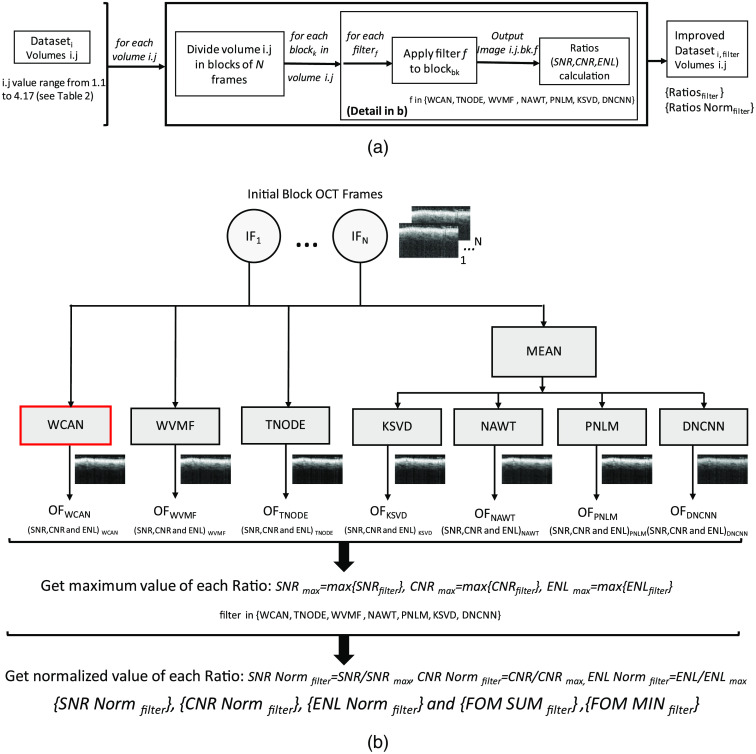
(a) Process used for the evaluation of all methods with all datasets. Each volume in the study was divided into blocks of N frames. For each block, all filters were applied and the evaluation metrics were computed. Finally, all blocks were combined and the improved version of each volume and each filter was created. (b) Details of the process for each block. The compounding of each block with N initial frames (${\mathrm{IF}}_{1},\ldots {\mathrm{IF}}_{N}$) was performed using WCAN, WVMF, TNODE, and MEAN. The output of the MEAN was applied to the methods KSVD, NAWT, PNLM, and DNCNN. The output frames from each method (${\mathrm{OF}}_{\mathrm{WCAN}}$, ${\mathrm{OF}}_{\mathrm{WVMF}}$, ${\mathrm{OF}}_{\mathrm{TNODE}}$, ${\mathrm{OF}}_{\mathrm{KSVD}}$, ${\mathrm{OF}}_{\mathrm{NAWT}}$, ${\mathrm{OF}}_{\mathrm{PNLM}}$, and ${\mathrm{OF}}_{\mathrm{DNCNN}}$), as applied to all frames, were used in the quantitative assessment. The normalized value of the metrics (*SNR Norm*, *CNR Norm*, *ENL Norm*, and *FOM*) were calculated with respect to the maximum value of each metric from the output frames. We used these normalized values for all volumes in comparing the quantitative assessments for all filters.

We compared the performance of the WCAN algorithm with respect to other state-of-the-art methods via two different approaches. First, we used 2D filters that have previously demonstrated excellent performance in OCT speckle reduction such as PNLM,^32^ complex wavelet based K-SVD (KSVD),^46^ and noise adaptive wavelet thresholding (NAWT)^43^ or in general image denoising (DNCNN).^34^ Next, we used two 3D filters: i.e., the wavelet multiframe algorithm (WVMF)^45^ and TNODE.^47^ Table 4 gives sources for implementations of these methods. Figure 4 shows the process of evaluation for the various methods. All steps were performed over all frames in every dataset. The denoised OCT frames generated as outputs were compared using the *SNR*, *CNR*, and *ENL* performance metrics.

**Table 4:** State-of-the-art denoising software used in the assessment of the proposed method.

Year	Denoising method	Official website
2012	WVMF^45^	Image denoising algorithms archive.
2015	KSVD^46^	State-of-the-art method for OCT denoising.
2016	PNLM^32^	PNLM: a probability-based non-local means filter for speckle noise suppression in optical coherence tomography images.
2017	DNCNN^34^	Beyond gaussian denoiser: residual learning of deep CNN for image denoising.
2017	NAWT^43^	Implemented following the authors’ manuscript.
2018	TNODE^47^	Volumetric non-local-means based speckle reduction for optical coherence tomography.

## 3 Results and Discussion

### 3.1 Parameter Assessment

**Fig. 5:**
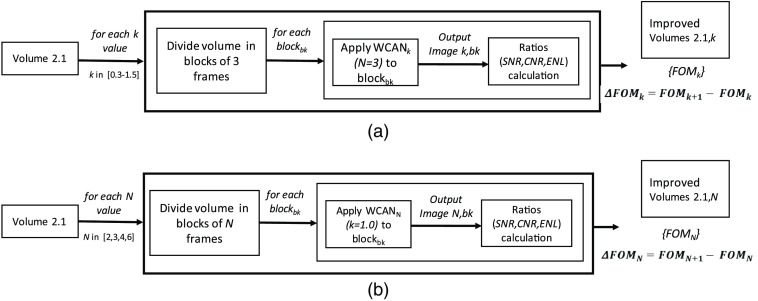
Process followed for the assessment of the parameters k and N using Volume 2.1 from the MUW-2 dataset and the reference frames from Volume R.2. (a) Evaluation of the parameter k with N=3 for values of k from 0.3 to 1.4 in steps of 0.1. We divided the volume into blocks of three frames and applied WCAN for each k value. After calculating the quantitative metrics, we compared the resulting *FOMs* of the enhanced frames for consecutives values of k. (b) Evaluation of the parameter N with k=1 for values of N from 2 to 6. For each N value, we divided the volume into blocks of N frames and applied WCAN with k set to 1. After calculating the quantitative metrics, we compared the resulting *FOM* of the enhanced frames for consecutives values of N.

Before applying the method to all datasets and comparing it with the state-of-the-art filters, we evaluated the impact of the two main parameters of the algorithm, i.e., k [the threshold parameter in Eq. (3)] and N (the number of frames to consider in the compounding process). We used Volume 2.1 (see Table 2) and the reference frames from Volume R.2 (Table 3) for this purpose in the two experiments outlined in Fig. 5.

First, we varied the parameter k from 0.3 to 1.5 in steps of 0.1, with N set to 3. In the second experiment, we varied the parameter N from 2 to 6, with k set to 1.0. After applying the WCAN method in both cases, we calculated the quantitative metrics (*SNR*, *CNR*, and *ENL*) and the resulting $FOM_{SUM}$ (only *FOM* for the rest of this section). We then computed the difference in the *FOM* values for each enhanced frame for two consecutive values of k (such as k=0.4 and 0.3) and N (such as N=3 and N=2). This process generated a set of *FOM*-value differences $\Delta FOM_{K}$ for each pair of consecutive k values and a set of $\Delta FOM_{N}$ for each pair of consecutive N values.

**Fig. 6:**
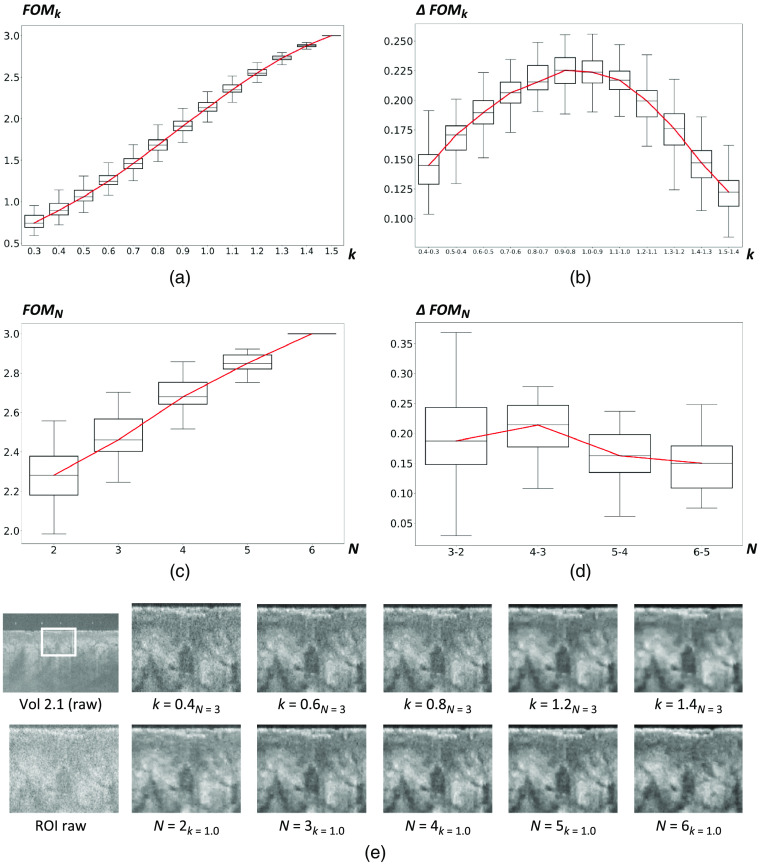
Results of the evaluation of the parameters k and N for the WCAN method, using the Volume 2.1 from the MUW-2 dataset and the reference frames from Volume R.2. (a) *FOM* for consecutive k values from 0.3 to 1.5. (b) Improvement in the *FOM* metric for consecutive k values from 0.3 to 1.5. The horizontal axis represents consecutive pairs of values for k. The vertical axis represents the *FOM* differences over all frames in the volume for consecutive pairs of values for k. (c) *FOM* for consecutive N values from 2 to 6. (d) Improvement in *FOM* metric for consecutive N values from N=2 to N=6. The horizontal axis represents consecutive pairs of values for N. The vertical axis represents the *FOM* differences over all frames in the volume for consecutives pairs of values for N. (e) Examples of the application of the WCAN method for different combinations of k and N in the ROI marked as a white box in the top-left raw noisy OCT image.

Figure 6 gives the main results and some examples of the application of the WCAN method to Volume 2.1. We observe that the improvement in the quantitative metrics reaches a maximum between k=0.8 and 0.9, with a mean improvement in *FOM* of 0.213, and any further increase in k decreases the incremental improvement. Note that this does not imply that k=0.9 is the optimal value to use but that k=0.9 is the value for which we have the greatest improvement with respect to the previous value of k. Although increasing the value of k always produces a quantitative improvement in the image, there are diminishing returns from increasing k beyond k=0.9. We can observe the same trend for the parameter N, the maximum mean improvement of *FOM*, i.e., $\Delta FOM_{N}=0.229$, occurs between N=4 and N=3. The image examples in Fig. 6 show how increases in N and k produce better noise reduction in the resultant image. One of the main advantages of the proposed method is that it is easily adaptable to very different OCT settings by simply adjusting these two parameters.

To find appropriate values of k and N for the quantitative assessment, we used the first volume in each dataset (Volume IDs 1.1, 2.1, 3.1, and 4.1 for the MUW-1, MUW-2, MUW-3, and A2ASDOCT datasets, respectively, in Table 2 and the references frames with Volumes IDs R.1, R.2, R3, and R.4 I in Table 3). Table 5 gives the final values chosen for each dataset, having been determined empirically as a suitable balance between performance metric improvement, qualitative visual quality, and execution time.

**Table 5:** Parameters used in the assessment of the WCAN method for each dataset. N is the number of frames considered at consecutive positions in the compounding process. k is the adjustment parameter for the thresholding process. The number of decomposition levels, L, was set to four for all datasets.

Parameter	MUW-1 dataset	MUW-2 dataset	MUW-3 dataset	A2ASDOCT dataset
N	3	3	2	4
k	1	1	1.1	1.6

We also evaluated the influence of the wavelet family in the calculation of 2D discrete stationary wavelet transform. We tested the wavelets families Haar, Daubechies (db1-db10), Symlets (sym2-sym8), Coiflets (coif1-coif5), and BiorSplines (bior1.1, bior1.3, bior1.5, bior2.2, bior2.4, bior2.6, bior2.8, bior3.1, bior3.3, bior3.5, bior3.7, bior3.9, bior4.4, bior 5.5, and bior6.8) using the Volume ID 2.1 and the reference set R.2 of Table 3. The Table 6 presents the results of the wavelet with the best performance in each family. As we can observe, the Haar wavelet shows the best performance and was the wavelet selected for the rest of the experiments.

**Table 6:** Quantitative evaluation of the wavelet families Haar, Daubenchies, Symlets, Coiflets and BiorSplines, considering the Volume ID 2.1 and the reference set R.2 from Table 3. The results show the mean ± standard deviation of the improvement with respect to the initial metrics presented in Table S1 in the Supplementary Material (raw images).

Wavelet	*SNR*	*CNR*	*ENL*
*haar*	12.30 ± 2.44	2.02 ± 0.36	622.80 ± 101.53
*db2*	11.78 ± 2.25	1.86 ± 0.31	537.78 ± 82.06
*sym3*	11.07 ± 2.03	1.85 ± 0.32	571.68 ± 79.25
*coef1*	11.74 ± 2.23	1.85 ± 0.31	533.41 ± 81.91
*bior3.3*	11.71 ± 2.21	1.82 ± 0.30	521.72 ± 81.37

### 3.2 Quantitative Evaluation

**Fig. 7:**
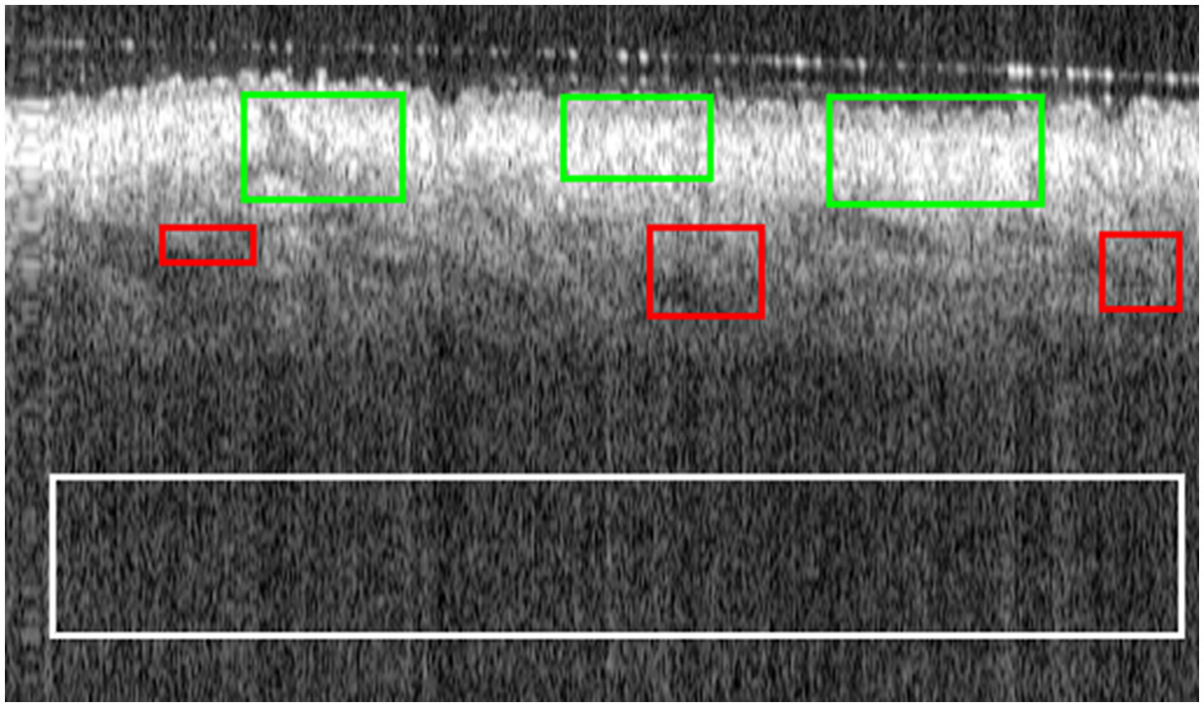
OCT raw image and ROIs used for the calculation of the quality metrics. The white rectangular region was used for the noise estimation; the red rectangles represent the homogeneous regions (H=3) used to calculate *ENL*, and the green rectangles represent the nonhomogeneous regions. The sum of both individual metrics is used to calculate the *CNR* (R=6).

**Fig. 8:**
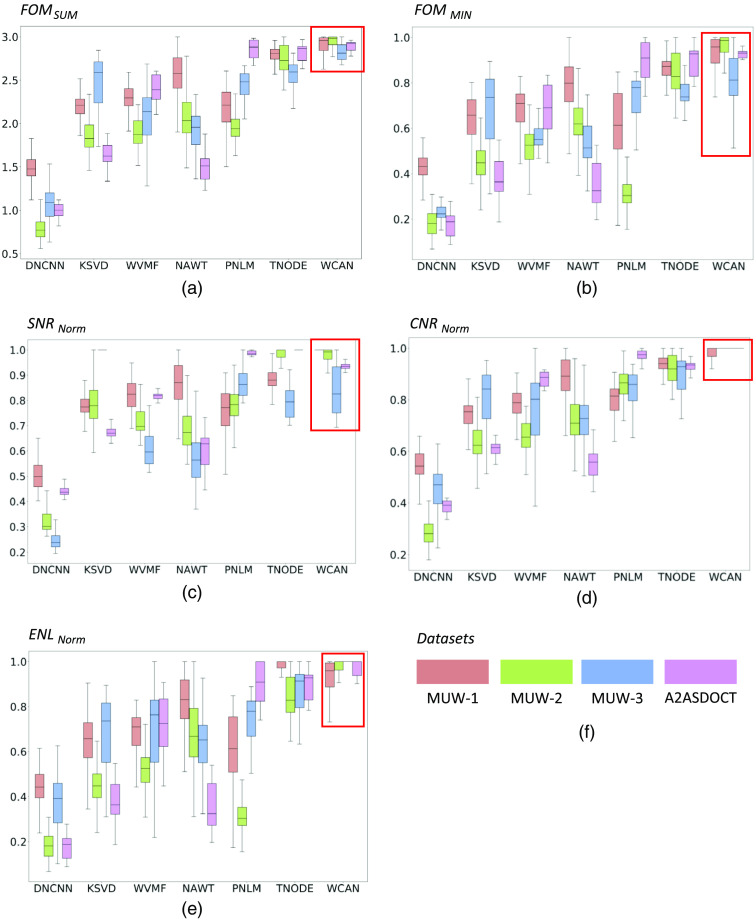
Value distribution for the different metrics of the quantitative evaluation of all datasets used in the study (see Table 2). The proposed WCAN method is shown in the rightmost column within the red box. (a) $FOM_{SUM}$ calculated using Eq. (14). (b) $FOM_{MIN}$ calculated using Eq. (15). (c) *SNR* *Norm* calculated using Eq. (11) and normalized with respect to the maximum value of the metric for each denoised frame. (d) *CNR Norm* calculated using Eq. (12) and normalized with respect to the maximum value of the metric for each denoised frame. (e) *ENL Norm* calculated using Eq. (13) and normalized with respect to the maximum value of the metric for each denoised frame. (f) Order of the datasets for each method.

We now present the results of our quantitative evaluation of the application of the compared methods to the complete stack of frames for all four datasets in this study. In the evaluation, we used the same ROIs for all frames in the same volume to maintain a consistent reference (Fig. 7 shows one frame with the ROIs used for Volume 1.1). The detailed values of the metrics for all datasets are included in Tables S2–S7 in the Supplementary Material. In Tables 7–10 and Fig. 8, we present the aggregate results. Tables 7–10 show the mean ± standard deviation of the improvement with respect to the initial metrics presented in Table S1 in the Supplementary Material (raw images). The values corresponding to the best performance for the individual metrics and for the *FOMs* are shown in bold.

**Table 7:** Quantitative evaluation for the MUW-1 dataset, considering the results of the application of all filters to Volumes 1.1–1.5 (see Table 2) and the reference frames R.1 (see Table 3). The compounding was performed using three B-scans per position.

Filter	*SNR Norm*	*CNR Norm*	*ENL Norm*	${FOM}_{SUM}$	${FOM}_{MIN}$
WCAN	**0.99 ± 0.026**	**0.97 ± 0.065**	0.91 ± 0.117	**2.87 ± 0.192**	**0.91 ± 0.117**
TNODE	0.88 ± 0.044	0.93 ± 0.057	**0.95 ± 0.101**	2.77 ± 0.148	0.86 ± 0.075
PNLM	0.75 ± 0.102	0.80 ± 0.067	0.61 ± 0.168	2.16 ± 0.271	0.61 ± 0.165
NAWT	0.87 ± 0.093	0.89 ±0.083	0.82 ±0.126	2.58 ± 0.262	0.80 ± 0.119
WVMF	0.82 ± 0.051	0.78 ± 0.064	0.68 ± 0.101	2.28 ± 0.166	0.68 ± 0.101
KSVD	0.80 ± 0.085	0.73 ± 0.064	0.64 ± 0.105	2.18 ± 0.169	0.64 ± 0.101
DNCNN	0.50 ± 0.051	0.54 ± 0.059	0.44 ± 0.077	1.48 ± 0.137	0.42 ± 0.066

**Table 8:** Quantitative evaluation for the MUW-2 dataset, considering the results of the application of all filters to Volumes 2.1–2.5 (see Table 2) and the reference frames R.2 (see Table 3). The compounding was performed using three B-scans per position.

Filter	*SNR Norm*	*CNR Norm*	*ENL Norm*	${FOM}_{SUM}$	${FOM}_{MIN}$
WCAN	0.97 ± 0.064	**0.98 ± 0.055**	**0.96 ± 0.075**	**2.91 ± 0.187**	**0.95 ± 0.076**
TNODE	**0.98 ± 0.026**	0.92 ± 0.055	0.85 ± 0.100	2.75 ± 0.165	0.85 ± 0.099
PNLM	0.78 ± 0.075	0.87 ± 0.055	0.31 ± 0.062	1.96 ± 0.142	0.31 ± 0.062
NAWT	0.68 ± 0.076	0.74 ± 0.116	0.69 ± 0.178	2.11 ± 0.335	0.62 ± 0.114
WVMF	0.72 ± 0.053	0.66 ± 0.057	0.52 ± 0.086	1.89 ± 0.153	0.52 ± 0.086
KSVD	0.78 ± 0.083	0.64 ± 0.080	0.45 ± 0.085	1.87 ± 0.213	0.45 ± 0.085
DNCNN	0.32 ± 0.048	0.29 ± 0.048	0.18 ± 0.052	0.79 ± 0.121	0.18 ± 0.052

**Table 9:** Quantitative evaluation for the MUW-3 dataset, considering the results of the application of all filters to Volumes 3.1–3.8 (see Table 2) and the reference frames R.3 (see Table 3). The compounding was performed using two B-scans per position.

Filter	*SNR Norm*	*CNR Norm*	*ENL Norm*	$FOM_{SUM}$	${FOM}_{MIN}$
WCAN	0.85 ± 0.102	**1.00 ± 0.027**	**0.99 ± 0.040**	**2.84 ± 0.112**	**0.84 ± 0.097**
TNODE	0.80 ± 0.063	0.90 ± 0.070	0.87 ± 0.103	2.56 ± 0.144	0.77 ± 0.045
PNLM	0.87 ± 0.055	0.84 ± 0.071	0.74 ± 0.104	2.46 ± 0.150	0.31 ± 0.052
NAWT	0.58 ± 0.116	0.72 ± 0.095	0.64 ± 0.128	1.95 ± 0.267	0.55 ± 0.092
WVMF	0.61 ± 0.066	0.75 ± 0.139	0.68 ± 0.194	2.05 ± 0.313	0.51 ± 0.068
KSVD	**0.98 ± 0.049**	0.81 ± 0.112	0.69 ± 0.156	2.48 ± 0.302	0.45 ± 0.077
DNCNN	0.25 ± 0.040	0.44 ± 0.115	0.37 ± 0.138	1.07 ± 0.254	0.18 ± 0.041

**Table 10:** Quantitative evaluation for the A2ASDOCT dataset, considering the results of the application of all filters to the 17 retinal sets (see Table 2) and the reference frame R.4 (see Table 3). The compounding was performed using four B-scans per position.

Filter	*SNR Norm*	*CNR Norm*	*ENL Norm*	$FOM_{SUM}$	${FOM}_{MIN}$
WCAN	0.93 ± 0.040	**1.00 ± 0.009**	**0.96 ± 0.065**	**2.89 ± 0.073**	**0.91 ± 0.057**
TNODE	0.99 ± 0.028	0.93 ± 0.024	0.90 ± 0.067	2.82 ± 0.098	0.89 ± 0.061
PNLM	**0.99 ± 0.009**	0.97 ± 0.026	0.90 ± 0.091	2.86 ± 0.112	0.89 ± 0.083
NAWT	0.60 ± 0.074	0.55 ± 0.060	0.35 ± 0.107	1.50 ± 0.171	0.35 ± 0.099
WVMF	0.81 ± 0.055	0.88 ± 0.027	0.70 ± 0.139	2.39 ± 0.155	0.67 ± 0.122
KSVD	0.67 ± 0.046	0.60 ± 0.037	0.38 ± 0.096	1.65 ± 0.135	0.38 ± 0.096
DNCNN	0.43 ± 0.044	0.38 ± 0.026	0.18 ± 0.058	1.00 ± 0.082	0.18 ± 0.059

To facilitate comparison between metrics, we used the normalized value of each metric, calculated from the maximum values for all filtered images in each group of frames [see Fig. 4(b)]. We can then refer to metric values corresponding to the best filtered output for each group of frames to make fair comparisons across the volume. We should first note that the best results across all datasets were accomplished by WCAN, with the highest values for $FOM_{SUM}$ and standard deviations between the smallest (MUW-3) and the second smallest (MUW-1, MUW-2, and A2ASDOCT). We have the same situation with the metric $FOM_{MIN}$, where WCAN presents the highest values. The TNODE algorithm had the second-best performance across all datasets. WCAN was also the best option, in most cases, when considering each metric separately. It returned the best *CNR* and *ENL* results for all datasets (except for MUW-1, where it had the second highest *ENL*). WCAN had the best *SNR* score for MUW-1, was second for MUW-2, and third for MUW-3 and A2ASDOCT.

Another notable aspect of the WCAN results is its consistent performance with very different OCT image settings, image samples, and locations on the skin (see Table 2) and retina. As is clear from Figs. 8(a) and 8(b), other filters such as PNLM and KSVD perform very differently for the retina dataset (A2ASDOCT) than for the skin datasets (MUW-1, MUW-2, and MUW-3).

**Fig. 9:**
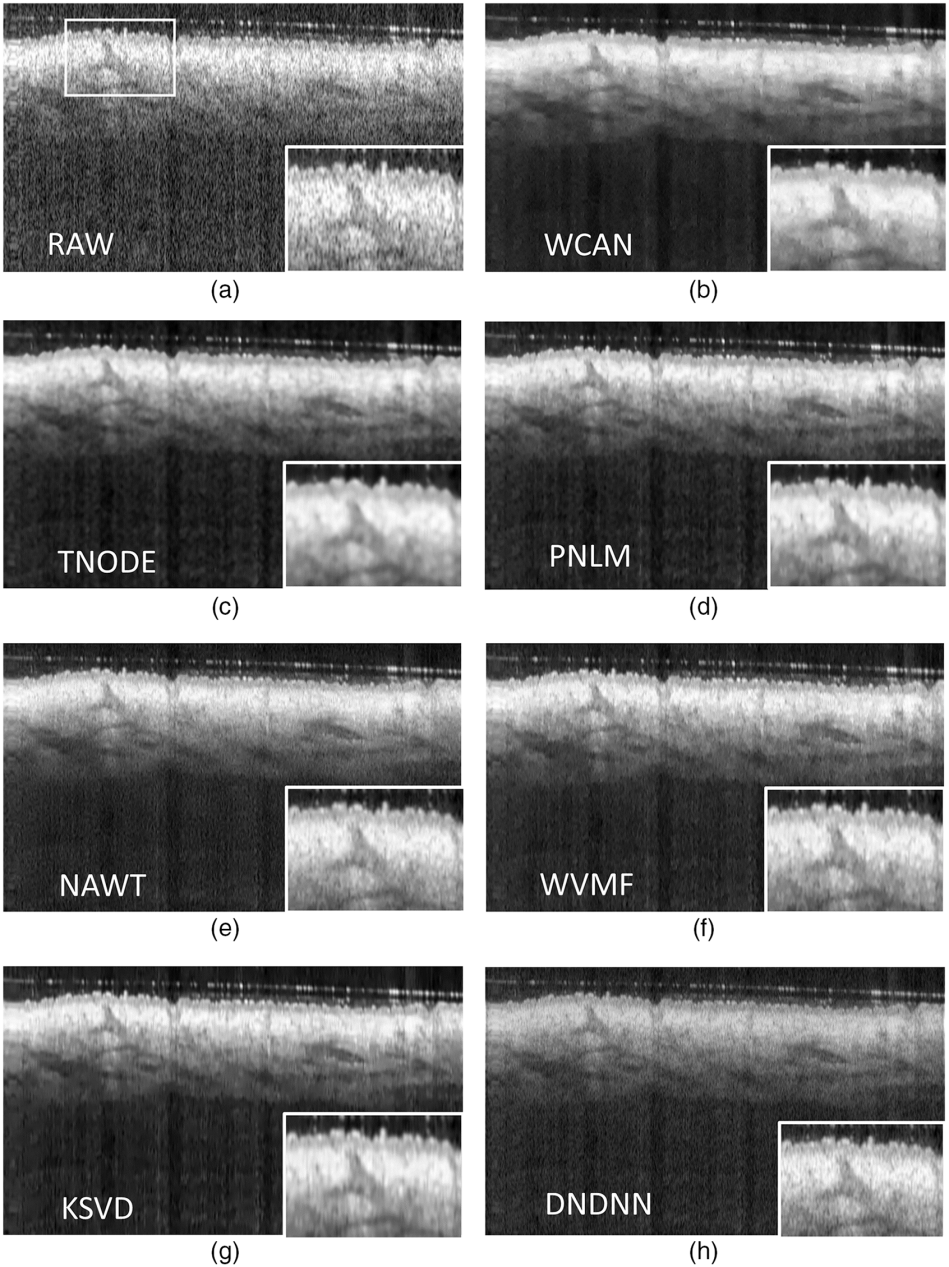
Comparison of the application of speckle reduction methods in a frame with 1100×512  pixels from Volume 1.1 using three B-scans at consecutive positions and the reference frames R.1. (a) Raw OCT image with a detailed ROI. The remainder of the image is the filtered OCT image after applying the method. (b) Proposed WCAN method. (c) TNODE method. (d) PNLM method. (e) NAWT method. (f) WVMF method. (g) KSVD method. (h) DNCNN method.

Figure 9 shows the significant differences in the OCT images generated by applying the state-of-the-art methods to Volume 1.1 and the reference frames R.1. Videos S1–S4 (see Figs. 13–16 in the Appendix) show the results of the application of the method over frames of the datasets MUW-1, MUW-2, MUW-3, and A2SDOCT.

Simple averaging was also evaluated. We used three volumes from datasets MUW-1, MUW-2, and MUW-3, where we have enough frames to perform the experiment in each volume. In particular, we considered Volumes ID 1.1, 2.1, 3.1 (Table 2) and the reference sets R.1, R.2, and R.3 of Table 3, respectively. We applied the WCAN method to each volume using the parameters previously identified in Table 5. In particular, we applied the WCAN to the three first frames of Volume ID 1.1 and 2.1 with k=1 and to the first two frames of Volume ID 3.1 with k=1.1. Then, we performed the average of N frames iteratively until the metric $FOM_{SUM}$ of averaging frames improved the metric of WCAN previously calculated. The results showed that to improve WCAN we needed to average 12, 27, and 13 frames for the Volumes ID 1.1, 2.1, and 3.1, respectively.

**Fig. 10:**
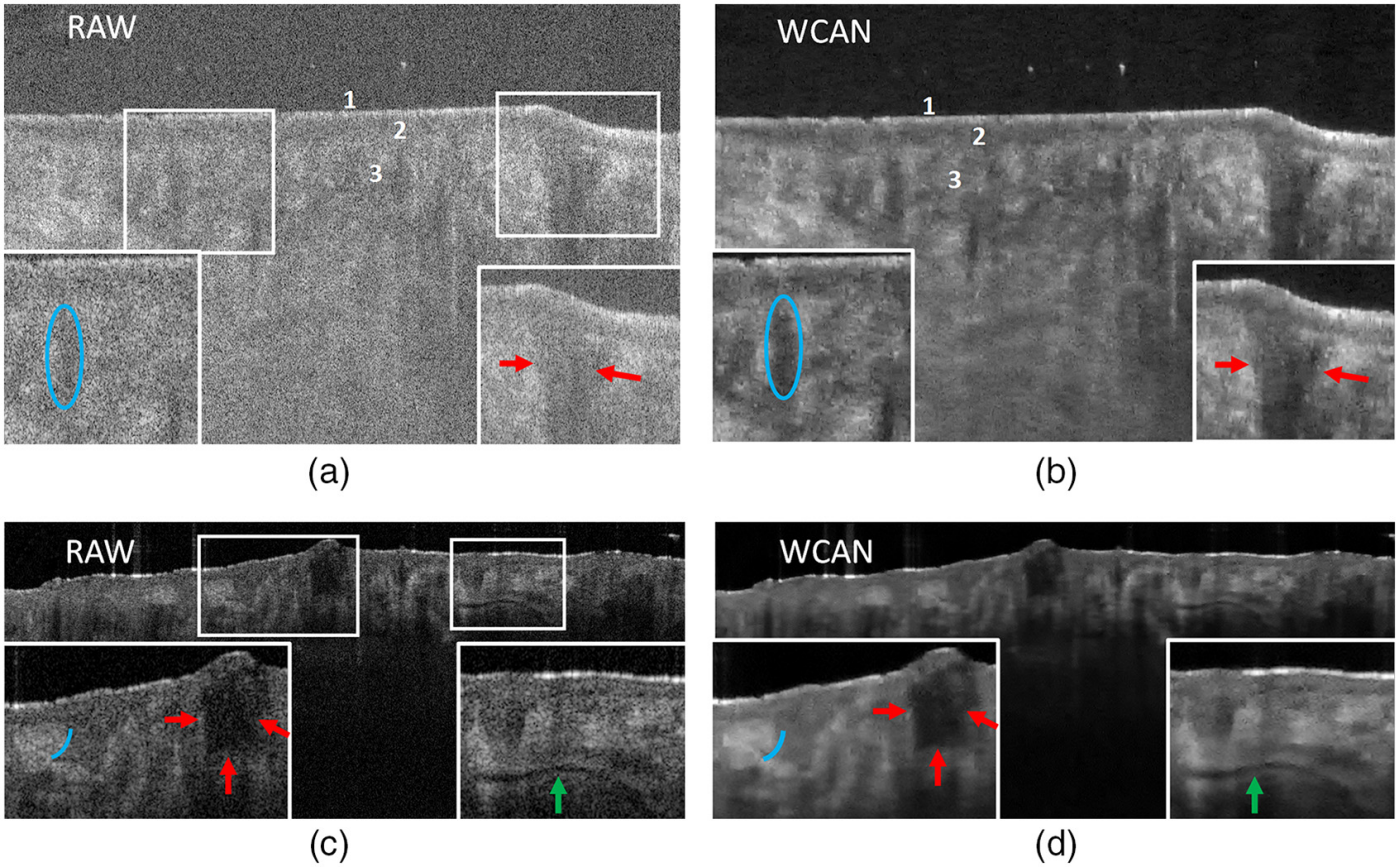
Comparison of the application of the WCAN method to frames from the MUW-2 and MUW-3 datasets. (a) Raw OCT skin image from a 1000×680  pixel sample of a head affected by BCC (Volume 2.4). Two detail ROIs are identified by white rectangular boxes. The digits 1, 2, and 3 in the image identify the stratum corneum, epidermis, and dermis layer, respectively. The blue ellipse and red arrows indicate regions that show the contrast enhancement obtained by filtering the raw image. (b) WCAN image processed using three consecutive frames from Volume 2.4, starting from the a frame. (c) Raw OCT skin image from a 1038×460  pixel sample of a cheek with folliculitis (Volume 3.8). Two zoom-in ROIs are identified by white rectangular boxes. The blue line and the red and green arrows identify different areas affected by speckle reduction after application of the proposed method. (d) WCAN image processed using two consecutive frames from Volume 3.8 starting from the c frame.

Figure 10 shows an analysis of some examples of the WCAN algorithm’s performance with skin images corresponding to frames of the MUW-2 and MUW-3 datasets. The first sample (Volume 2.4) provides information about a superficial BCC on the head. In Figs. 10(a) and 10(b), speckle reduction using WCAN [Fig. 10(b)] enables a stronger identification of the skin layers (marked with digits 1–3) than does the raw image [Fig. 10(a)]. In addition, the stratum corneum layer and the epidermis–dermis interface are more clearly distinguished. The boundaries and contrast are also enhanced, shown by comparing the area enclosed by the blue ellipse and the red arrows in the zoom-in boxes in the raw image [Fig. 10(a)] and after filtering [Fig. 10(b)]. The second example (Volume 3.8) shows a cheek with folliculitis. Figures 10(c) and 10(d) show a sharper difference between the regions indicated by the blue line and the red arrows and the structure indicated by the green arrow in the zoom-in boxes.

**Fig. 11:**
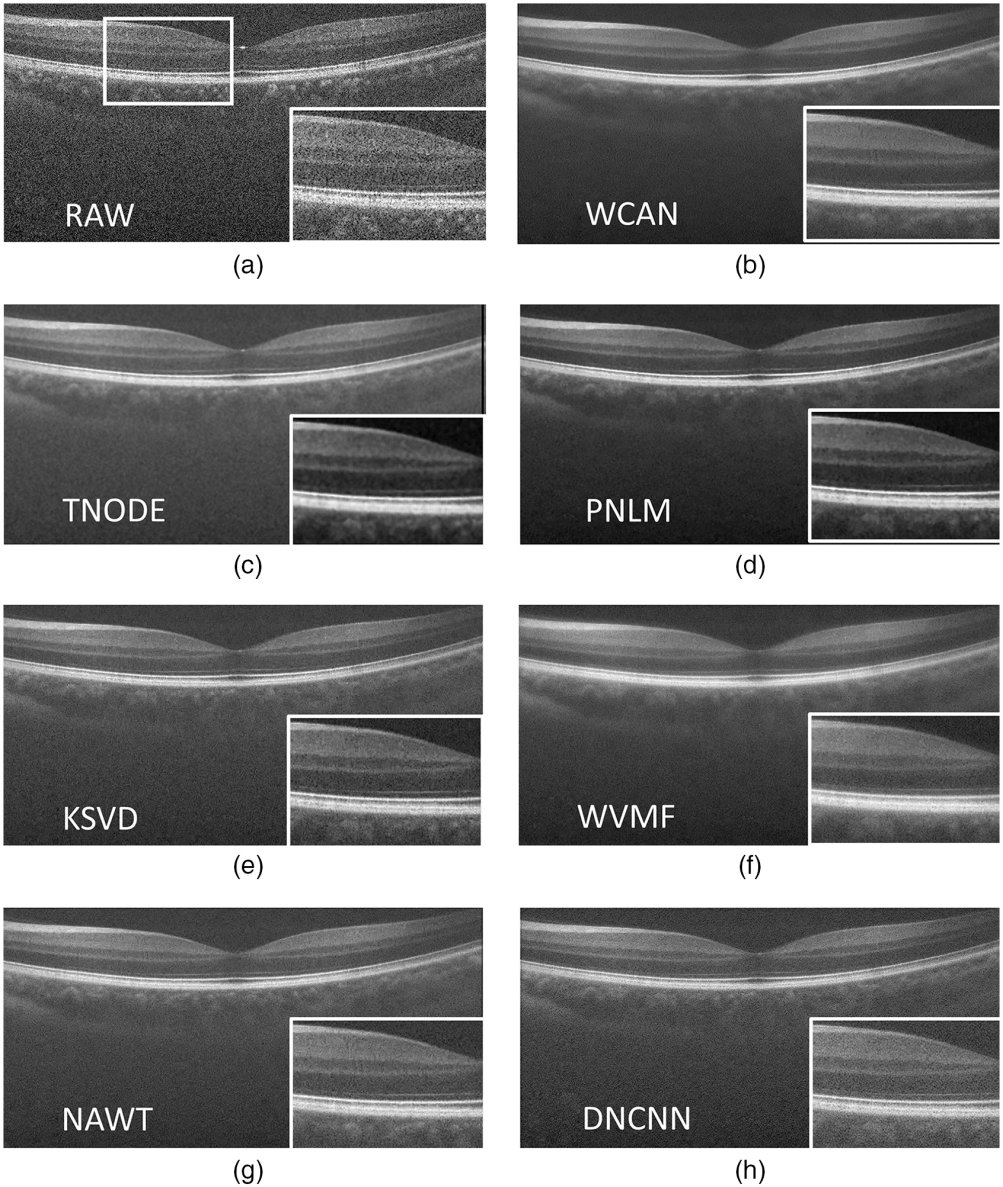
Comparison of the application of speckle reduction methods in a 900×450-pixel frame from Volume 4.1 using four B-scans at consecutive positions. (a) Raw OCT image and a detail ROI used for the qualitative comparison of all methods. (b) Proposed WCAN method. (c) TNODE method. (d) PNLM method. (e) KSVD method. (f) WVMF method.

Figure 11 enables qualitative comparisons for a retinal image from the A2ASDOCT database after applying each of the denoising methods in the study. Figure 11 shows that the WCAN algorithm gives significant noise suppression, which enables clear identification of the boundaries between retinal layers.

**Fig. 12:**
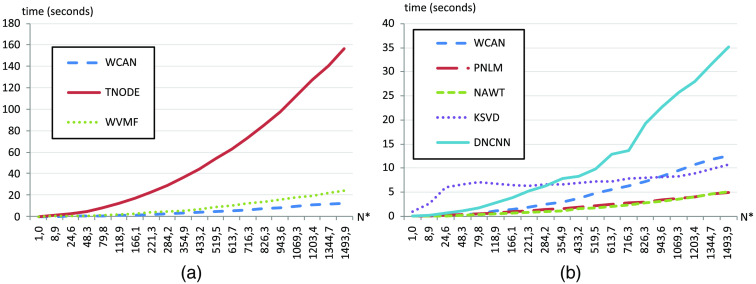
Comparison of the computation time in seconds of all methods of the study (WCAN, TNODE, WVMF, PNLM, NAWT, KSVD, and DNCNN). The parameter $N^{*}$ represents the total number of the pixels considered in each test (1100×512×2×scale) normalized with respect to the smallest one (1100×512×2×0.1). (a) Computation time comparison of WCAN with respect to the compounding methods TNODE and WVMF. (b) Computation time comparison of WCAN with respect to PNLM, NAWT, KSVD, and DNCNN methods.

Finally, to assess the computation time for the various algorithms we applied all methods of the study to 20 scaled versions of the first two frames of Volume ID 1.1. For the WCAN method we used k=1 and the reference frames R.1 from Table 3. The original size of the volume was two frames with 1100×512  pixels. We used scales from 0.1 to 4 in steps of 0.2 (20 scales in total) to resize the frames. For each scale we applied all of the methods and calculated the execution time using the method *timeit* of Matlab. In Fig. 12 we present the results. As shown in Fig. 12(a) the application of the WCAN method requires less execution time than the other pure compounding alternatives such as TNODE and WVMF. The PNLM and NAWT methods presented the best performance among all of the algorithms tested [Fig. 12(b)]. Finally, to estimate the computational complexity of the algorithm, we applied a basic fitting of the performance metrics of WCAN, resulting a linear polynomial function of the type y=p1*x+p2 with $p_{1}=0.0087$, $p_{2}=0.0086$, and the norm of residuals equaling 0.699. So we can estimate that the time complexity of the method is linear. The tests were executed in Matlab 2018b (MathWorks, Inc.) on a personal computer (Intel 3.3 GHz CPU, 32 GB memory).

## 4 Conclusion

This paper has described a speckle reduction method for OCT volumes. The method is based on a multiscale adaptive noise-wavelet-compounding strategy. The method requires the estimation of the noise variance in the wavelet domain of the OCT setting. In this study, we used previously acquired images of homogeneous scattering examples. The noise variance is used to compute the weights for the detail coefficients of each subband of each frame in the volume before compounding all of the filtered frames.

One of the major highlights of our method, and a novel contribution in the field of wavelet denoising, is the introduction of a new compounding scheme that integrates the noise variance influence of the input frames in relation to the variance of a set of reference frames. With this new approach, a visual inspection of the denoised frames presented shows the potential of the proposed algorithm to reduce the speckle noise efficiently without generating relevant denoising artifacts or producing a degradation of the structures present in the image. The simplicity, the ability of the method to adapt to different environments (OCT settings and noise levels), the robustness above all metrics considered, and its reduced computational time are its most significant advantages. Another potential benefit is the capacity of the method to adjust the amount of noise reduction by the modification of the parameter k, which may facilitate the adaptation to different clinical needs. The main weaknesses of the method is the need for a prior acquisition of a homogeneous sample to determine the noise variance used in the adaptive compounding of the detail coefficients in the wavelet decomposition. Nevertheless, as described in the estimation of the noise in datasets MUW-2 and A2ASDOCT, this drawback can be avoided through the use of a homogeneous ROI of the input frames. The other line of improvement is the automatic assessment of the parameters of the method (k and the number of frames of the input and reference frames). However, this assessment is needed only once when fine tuning the algorithm in a particular OCT setting.

A thorough evaluation was performed by applying the method to four different OCT settings, i.e., around 18 skin volumes in three datasets and 17 retinal volumes in a fourth dataset. We compared our method’s results with those from six state-of-the-art algorithms. The results of a quantitative evaluation based on five different metrics demonstrated that the proposed method achieved the best performance among the tested techniques in suppressing noise. A qualitative visual comparison of images confirmed the relative performance of the proposed method and suggests the potential application of the proposed OCT denoising technique to improving image quality prior to clinical diagnosis based on visual assessment. Future work will involve an automatic process for the assessment of the parameters of the method (k and the number of frames of the input and reference frames) through the training of a machine learning model based on quantitative metrics. Furthermore, a formal qualitative evaluation will be performed by clinical experts tackling the specific needs in their clinical routine, such as the capacity of the method to preserve or enhance the identification of tissue layers and their borders and the ability to visualize structures or to identify vasculatures.

## 5 Appendix

**Fig. 13:**
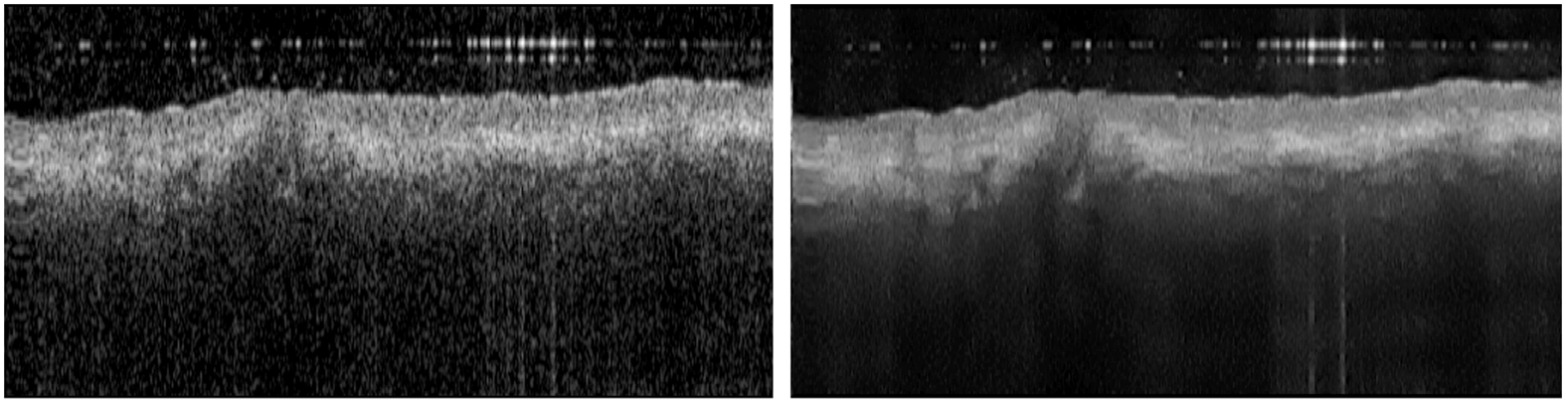
Results of the application of the WCAN method over frames from dataset MUW-1. Volumes 1.1–1.5 (see Table 2). using three B-scans at consecutive positions. (a) OCT raw image Volume 1.2 (b) WCAN image.(Video S1, MP4, 8.3MB [URL: https://doi.org/10.1117/1.JBO.26.6.065001.1]).

**Fig. 14:**
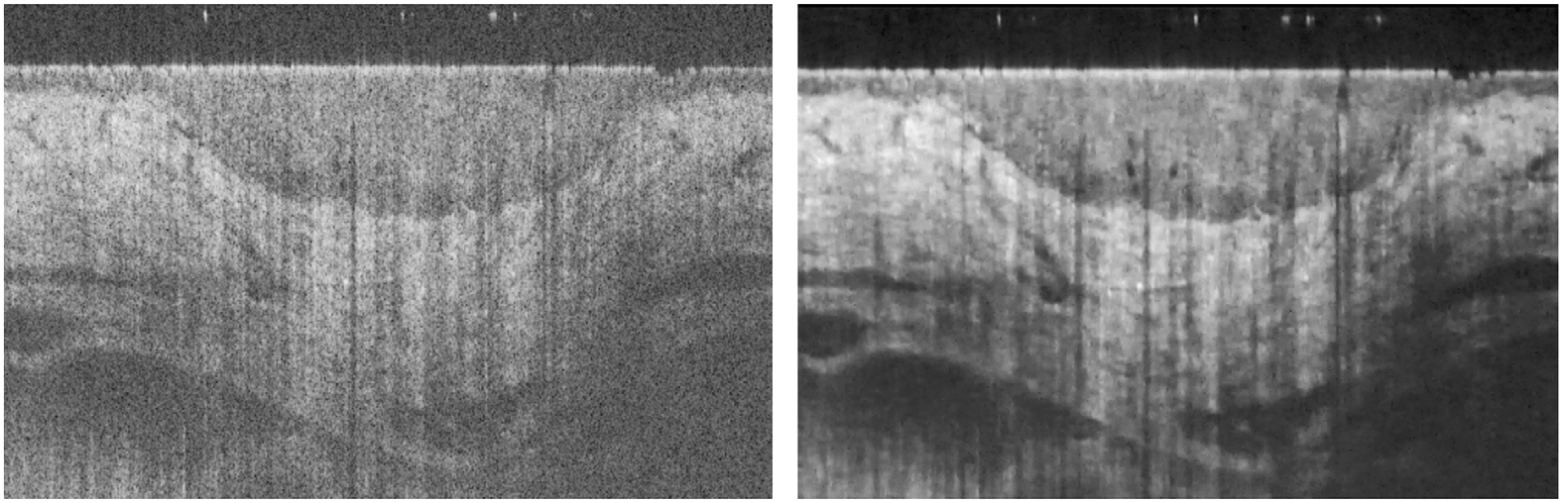
Results of the application of the WCAN method over frames from dataset MUW-2, Volumes 2.1–2.5 (see Table 2), using three B-scans at consecutive positions. (a) OCT raw image Volume 2.5 (b) WCAN image. (Video S2, MP4, 10.6 MB [URL: https://doi.org/10.1117/1.JBO.26.6.065001.2]).

**Fig. 15:**
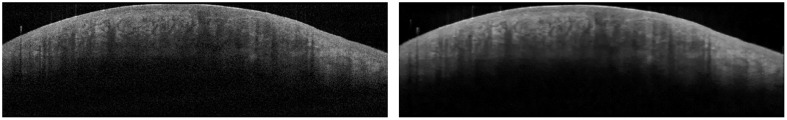
Results of the application of the WCAN method over frames from dataset MUW-3, Volumes 3.1–3.5 (see Table2), using two B-scans at consecutive positions. (a) OCT raw image Volume 3.3 (b) WCAN image. (Video S3, MP4, 1.8 MB [URL: https://doi.org/10.1117/1.JBO.26.6.065001.3]).

**Fig. 16:**
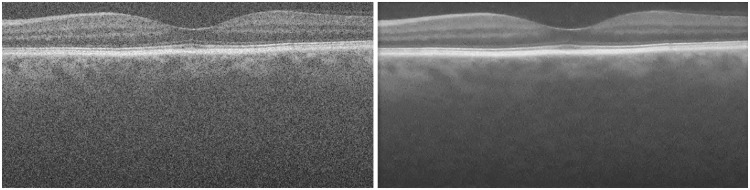
Results of the application of the WCAN method over frames from dataset A2ASDOCT, Volumes 4.1–4.17 (see Table 2), using four B-scans at consecutive positions. (a) OCT raw image Volume 4.12 (b) WCAN image. (Video S4, MP4, 9.9 MB [URL: https://doi.org/10.1117/1.JBO.26.6.065001.4]).

Videos S1–S4 (see Figs. 13–16) show the results of the application of the method over frames of the datasets MUW-1, MUW-2, MUW-3, and A2SDOCT.

## Code, Data, and Materials Availability

We have made a Matlab implementation of the WCAN method available through Code Ocean, including the full code for the noise estimation and the denoising algorithm, together with raw volume data for testing purposes.
