# Supplementary material for: Adaptive compounding speckle-noise-reduction filter for optical coherence tomography images
Source: J Biomed Opt. 2021 Jun 17;26(6):065001. doi: 10.1117/1.JBO.26.6.065001 (PMC8211087; doi:10.1117/1.JBO.26.6.065001)

Supplementary Material

Adaptive compounding speckle-noise-reduction filter for optical coherence tomography images

Juan J. Gómez-Valverde,[a,b*] Christoph Sinz,[c] Elisabet A. Rank,[d] Zhe Chen,[d] Andrés Santos,[a,b] Wolfgang Drexler,[d] María J. Ledesma-Carbayo[a,b]
[a]Universidad Politécnica de Madrid , ETSI Telecomunicación , Biomedical Image Technologies Laboratory (BIT), ,28040 Madrid, Spain
[b]Biomedical Research Center in Bioengineering, Biomaterials and Nanomedicine (CIBER-BBN),Spain
[c]Medical University of Vienna, Department of Dermatology, Waehringer Guertel 18-20, AKH 7J, 1090, Vienna, Austria
[d]Medical University of Vienna , Center for Medical Physics and Biomedical Engineering, Waehringer Guertel 18-20, AKH 4L, 1090, Vienna, Austria

In this supplementary document, we provide the initial values of the metrics for all the volumes considered (Table S1) and the improvement in SNR (Table S2 and Table S5), CNR (Table S3 and Table S6) and ENL (Table S4 and Table S7) for datasets MUW-1, MUW-2, MUW-3 and A2ASDOCT. Furthermore, we include the reference to the videos (Figures S1-S4) with examples of the application of the method WCAN over all the volumes.

Table S1 Mean and standard deviation of the initial metrics (raw images) for the MUW-1, MUW-2, MUW-3 and A2ASDOCT datasets (Table 2).

Volume ID	*SNR(dB)*	*CNR*	*ENL*
**MUW-1 Dataset**			
1.1	13.51 ± 0.12	2.50 ± 0.19	21.615 ± 3.5
1.2	10.27 ± 0.37	2.406 ± 0.17	26.39 ± 5.4
1.3	13.77 ± 0.29	2.09 ± 0.15	51.72 ± 19.32
1.4	6.53 ± 0.45	1.57 ± 0.10	7.91 ± 0.64
1.5	8.84 ± 0.16	1.88 ± 0.12	12.49 ± 1.15
**MUW- 2 Dataset**			
2.1	17.11 ± 0.30	1.10 ± 0.01	60.802 ± 2.06
2.2	17.21 ± 0.06	1.62 ± 0.04	69.87 ± 3.28
2.3	17. 25 ± 0.08	1.12 ± 0.04	50.51 ± 1.94
2.4	17.10 ± 0.09	0.96 ± 0.07	53.78 ± 2.10
2.5	17.54 ± 0.16	1.68 ± 0.10	63. 79 ± 6.13
**MUW-3 Dataset**			
3.1	9.81 ± 1.18	2.08 ± 0.33	19.29 ± 6.82
3.2	10.28 ± 1.28	2.45 ± 0.14	16.97 ± 1.98
3.3	10.58 ± 1.33	2.45 ± 0.40	17.37 ± 5.75
3.4	9.88 ± 1.34	1.98 ± 0.24	14.29 ± 3.72
3.5	8.43 ±1.26	1.77 ± 0.13	10.98 ± 2.06
3.6	9.46 ± 1.35	1.87 ± 0.26	11.22 ± 3.83
3.7	9.69 ± 1.32	2.42 ± 0.23	20.37 ± 6.45
3.8	8.16 ± 1.01	1.76 ± 0.11	15.43 ± 2.38
**A2ASDOCT Dataset**			
4.1	8.22 ± 0.04	0.82 ± 0.02	11.87 ± 0.29
4.2	8.05 ± 0.05	0.68 ± 0.03	10.56 ± 0.21
4.3	8.78 ± 0.06	1.25 ± 0.03	10.56 ± 0.21
4.4	8.46 ± 0.02	1.01 ± 0.02	12.55 ± 0.52
4.5	7.57 ± 0.01	0.64 ± 0.01	9.56 ± 0.12
4.6	8.30 ± 0.16	0.75 ± 0.26	10.87 ± 0.41
4.7	9.31 ± 0.06	1.08 ± 0.03	15.75 ± 0.44
4.8	9.59 ± 0.05	0.86 ± 0.02	10.22 ± 0.36
4.9	6.03 ± 0.45	0.87 ± 0.02	7.18 ± 0.24
4.10	8.25 ± 0.07	1.53 ± 0.01	17.48 ± 0.62
4.11	6.90 ± 0.04	0.75 ± 0.13	7.79 ± 0.14
4.12	8.39 ± 0.04	0.94 ± 0.01	13.11 ± 0.12
4.13	8.60 ± 0.18	1.02 ± 0.01	14.61 ± 0.72
4.14	7.95 ± 0.05	0.87 ± 0.01	12.26 ± 0.32
4.15	8.14 ± 0.07	0.73 ± 0.01	11.26 ± 0.21
4.16	8.50 ± 0.05	0.99 ± 0.01	13.39 ± 0.26
4.17	7.85 ± 0.05	0.72 ± 0.02	10.87 ± 0.26

Table S2 *SNR* (dB) improvement for the MUW-1 (Volume IDs 1.X), MUW-2 (Volume IDs 2.X) and MUW-3 (Volume IDs 3.X) datasets. The volumes from the datasets MUW-1 and MUW-2 include 256 frames and were compounded using 3 consecutive frames. The volumes from the dataset MUW-3 include 50 frames and were compounded using 2 consecutive frames. The results show the mean ± standard deviation of the improvement with respect to the initial metrics presented in Table S1 (raw images). The best *SNR* in all the volumes are highlighted in bold. WCAN presented the best *SNR* improvement in all the volumes in the dataset MUW-1, in Volumes 2.1 and 2.2 in the dataset MUW-2 and in Volume 3.1 and 3.8 in dataset MUW-3. In the rest of the volumes WCAN presented the second score after TNODE (Volumes 2.3, 2.4 and 2.5) and after PNLM (Volumes 3.2 to 3.7).

Vol. ID	MEAN	DNCNN	KSVD	WVMF	NAWT	PNLM	TNODE	WCAN
1.1	3.88 ± 0.15	4.70 ± 0.15	8.30 ± 0.51	8.21 ± 0.39	7.84 ± 0.41	7.68 ± 0.52	9.39 ± 0.52	**10.76 ± 0.77**
1.2	3.18 ± 0.34	3.79 ± 0.39	6.80 ± 1.21	6.14 ± 0.86	6.43 ± 1.02	6.08 ± 0.88	6.25 ± 0.88	**6.94 ± 1.02**
1.3	3.26 ± 0.21	3.98 ± 0.23	5.61 ± 0.39	6.30 ± 0.46	7.16 ± 0.56	4.36 ± 0.50	6.92 ± 0.50	**7.23 ± 0.56**
1.4	3.13 ± 0.54	3.67 ± 0.66	5.89 ± 1.29	6.03 ± 1.26	6.72 ± 1.61	5.92 ± 1.29	6.33 ± 1.37	**7.64 ± 1.82**
1.5	3.91 ± 0.20	4.61 ± 0.22	7.13 ± 0.49	7.78 ± 0.50	7.93 ± 0.68	7.67 ± 0.56	8.38 ± 0.62	**9.45 ± 0.81**
2.1	4.34 ± 0.36	4.49 ± 0.35	10.24 ± 1.30	9,38 ± 1.30	8.93 ± 1.31	9.14 ± 2.52	12.38 ± 2.31	**12.45 ± 2.51**
2.2	4.70 ± 0.07	4.80 ± 0.07	12.59 ± 0.32	11.16 ± 0.32	10.05 ± 0.31	13.22 ± 1.14	15.81 ± 0.80	**16.50 ± 1.04**
2.3	4.73 ± 0.04	4.82 ± 0.04	13.03 ± 0.08	11.37 ± 0.08	11.33 ± 0.57	14.02 ± 0.65	**16.55 ± 0.23**	15.91 ± 0.50
2.4	4.70 ± 0.05	4.80 ± 0.05	11.01 ± 0.20	11.14 ± 0.20	10.22 ± 0.37	12.94 ± 0.79	**15.88 ± 0.53**	14.93 ± 2.29
2.5	4.36 ± 0.21	4.58 ± 0.18	10.81 ± 0.81	9.70 ± 0.81	9.69 ± 0.60	9.18 ± 1.68	**12.47 ± 1.43**	12.11 ± 1.31
3.1	3.55 ± 0.21	3.80 ± 0.20	11.68 ± 0.59	8.69 ± 0.59	10.35 ± 1.16	11.17 ± 1.17	10.56 ± 0.86	**12.30 ± 0.95**
3.2	3.99 ± 0.24	4.22 ± 0.24	**19.74 ± 0.27**	10.63 ± 0.27	8.72 ± 0.73	16.21 ± 0.25	14.31 ± 0.20	14.70 ± 0.38
3.3	3.90 ± 0.18	4.11 ± 0.19	**15.28 ± 0.73**	9.79 ± 0.73	8.66 ± 0.68	13.69 ± 1.84	12.59 ± 1.42	12.96 ± 1.63
3.4	4.07 ±0.11	4.29 ± 0.12	**16.78 ± 0.27**	10.26 ± 0.27	10.25 ± 0.39	14.50 ± 0.75	13.27 ± 0.53	13.51 ± 0.57
3.5	4.05 ± 0.17	4.25 ± 0.18	**19.68 ± 0.20**	10.67 ± 0.20	10.08 ± 0.42	15.88 ± 0.19	14.29 ± 0.09	14.23 ± 0.20
3.6	3.96 ± 0.47	4.15 ± 0.48	**18.54 ± 0.68**	10.44 ± 0.68	8.78 ± 0.98	15.34 ± 1.46	14.01 ± 1.10	14.28 ±1.42
3.7	4.01 ± 0.11	4.22 ± 0.12	**17.05 ± 0.21**	10.26 ± 0.21	10.13 ± 0.72	14.79 ± 0.69	13.44 ± 0.46	14.23 ± 0.47
3.8	3.71 ± 0.17	3.82 ± 0.18	14.32 ± 0.18	10.13 ± 0.18	9.43± 0.63	15.45 ± 0.19	14.13 ± 0.11	**16.08 ± 0.74**

Table S3 *CNR* improvement for the MUW-1 (Volume IDs 1.X), MUW-2 (Volumes IDs 2.X) and MUW-3 (Volume IDs 3.X) datasets. The volumes from the datasets MUW-1 and MUW-2 include 256 frames and were compounded using 3 consecutive frames. The volumes from the dataset MUW-3 include 50 frames and were compounded using 2 consecutive frames. The results show the mean ± standard deviation of the improvement with respect to the initial metrics presented in Table S1 (raw images). The best *CNR* in all the volumes are highlighted in bold. WCAN presented the best *CNR* improvement in all the volumes except in Volumes 1.2 and 2.3 were was the second after TNODE.

Vol. ID	MEAN	DNCNN	KSVD	WVMF	NAWT	PNLM	TNODE	WCAN
1.1	0.53 ± 0.10	0.69 ± 012	0.99 ± 0.21	0.97 ± 0.19	1.04 ± 0.19	1.07 ± 0.22	1.20 ± 0.25	**1.27 ± 0.28**
1.2	0.64 ± 0.12	0.88 ± 0.15	1.21 ± 0.27	1.30 ± 0.28	1.39 ± 0.24	1.20 ± 0.27	**1.65 ± 0.36**	1.59 ± 0.36
1.3	0.51 ± 0.13	0.69 ± 0.16	0.97 ± 0.29	1.02 ± 0.30	1.45 ± 0.44	1.13 ± 0.34	1.27 ± 0.38	**1.31 ± 0.41**
1.4	0.38 ± 0.07	0.48 ± 0.08	0.65 ± 0.12	0.72 ± 0.14	0.79 ± 0.17	0.71 ± 0.13	0.83 ± 0.17	**0.89 ± 0.18**
1.5	0.35 ± 003	0.44 ± 0.04	0.56 ± 0.06	0.61 ± 0.06	0.66 ± 0.05	0.61 ± 0.06	0.68 ± 0.06	**0.73 ± 0.07**
2.1	0.48 ± 0.04	0.55 ± 0.05	1.35 ± 0.15	1.26 ± 0.15	1.38 ± 0.23	1.82 ± 0.29	1.91 ± 0.31	**2.07 ± 0.39**
2.2	0.64 ± 0.04	0.73 ± 0.04	1.57 ± 0.12	1.60 ± 0.12	1.79 ± 0.13	2.01 ± 0.16	2.14 ± 0.18	**2.49 ± 0.23**
2.3	0.51 ± 0.04	0.56 ± 0.04	1.32 ± 0.13	1.47 ± 0.09	1.63 ± 0.17	2.04 ± 0.17	**2.28 ± 0.15**	2.28 ± 0.23
2.4	0.47 ± 0.03	0.52 ± 0.03	1.17 ± 0.12	1.30 ± 0.07	1.26 ± 0.07	1.73 ± 0.11	1.78 ± 0.11	**1.80 ± 0.35**
2.5	0.62 ± 0.05	0.72 ± 0.06	1.46 ± 0.16	1.45 ± 0.16	1.89 ± 0.25	1.75 ± 0.21	1.86 ± 0.23	**2.02 ± 025**
3.1	0.38 ± 0.19	0.50 ± 0.22	1.10 ± 0.66	0.93 ± 0.54	1.24 ± 0.65	1.23 ± 0.74	1.31 ± 0.77	**1.66 ± 1.04**
3.2	0.50 ± 0.13	0.61 ± 0.12	1.10 ± 0.29	1.05 ± 0.13	0.85 ± 0.15	1.15 ± 0.28	1.27 ± 0.27	**1.29 ± 0.27**
3.3	0.43 ± 0.21	0.52 ± 0.23	0.89 ± 0.44	0.90 ± 0.51	0.76 ± 0.39	0.96 ± 0.52	1.05 ± 0.57	**1.11 ± 0.61**
3.4	0.44 ± 0.12	0.53 ± 0.13	1.08 ± 0.35	1.01 ± 0.32	0.93 ± 0.27	1.05 ± 0.35	1.11 ± 0.37	**1.24 ± 0.39**
3.5	0.42 ± 0.07	0.50 ± 0.08	0.95 ± 0.19	0.87 ± 0.18	0.82 ± 0.13	0.94 ± 0.18	0.99 ± 0.18	**1.06 ± 0.19**
3.6	0.40 ± 0.14	0.46 ± 0.16	0.84 ± 0.26	0.84 ± 0.28	0.69 ± 0.26	0.84 ± 0.27	0.88 ± 0.28	**0.92 ± 0.30**
3.7	0.48 ± 0.08	0.58 ± 0.08	0.98 ± 0.16	0.96 ± 0.20	0.93 ± 0.15	1.01 ± 0.16	1.08 ± 0.16	**1.16 ± 0.17**
3.8	0.40 ± 0.05	0.49 ± 0.05	1.16 ± 0.18	1.03 ± 0.18	1.01 ± 0.18	1.38 ± 0.19	1.45 ± 0.15	**1.81 ± 0.20**

Table S4 *ENL* improvement for the MUW-1 (Volume IDs 1.X), MUW-2 (Volume IDs 2.X) and MUW-3 (Volumes IDs 3.X) datasets. The volumes from the datasets MUW-1 and MUW-2 include 256 frames and were compounded using 3 consecutive frames. The volumes from the dataset MUW-3 include 50 frames and were compounded using 2 consecutive frames. The results show the mean ± standard deviation of the improvement with respect to the initial metrics presented in Table S1 (raw images). The best *ENL* in all the volumes are highlighted in bold. WCAN presented the best *ENL* improvement in Volume 1.1 and all the volumes in the MUW-2 and MUW-3 datasets, except in Volume 2.5 where was the second after NAWT. WCAN was the second in Volumes 1.2 and 1.4 after TNODE, and the third in Volume 1.3 after NAWT and TNODE.

Vol. ID	MEAN	DNCNN	KSVD	WVMF	NAWT	PNLM	TNODE	WCAN
1.1	13.69 ± 3 .80	18.66 ± 4.94	29.11 ± 9.47	27.99 ± 8.92	32.44 ± 9.80	23.39 ± 9.47	40.13 ± 14.87	**41.45 ± 16.02**
1.2	19.18 ± 6.17	28.24 ± 8.90	39.02 ± 15.53	45.83 ± 17.66	48.16 ± 15.16	41.02 ± 17.04	**71.51 ± 31.13**	61.32 ± 25.88
1.3	38.44 ± 22.65	54.57 ± 29.09	94.48 ± 71.08	85.76 ± 54.15	**156.23 ± 98.35**	68.84 ± 55.01	132.17 ± 86.96	130.51 ± 92.74
1.4	6.04 ± 1.03	7.22 ± 1.22	10.78 ± 2.20	12.66 ± 2.76	13.46 ± 2.98	12.76 ± 2.75	**17.23 ± 3.98**	16.42 ± 3.83
1.5	5.22 ± 0.86	6.10 ± 0.98	8.71 ± 1.44	9.14 ± 1.52	10.17 ± 1.53	9.59± 1.56	**11.72 ± 1.86**	11.48 ± 1.92
2.1	70.06 ± 4.40	86.08 ± 5.45	286.50 ± 64.43	262.80 ± 23.07	354.06 ± 48.90	204.35 ± 33.69	535.31 ± 53.92	**660.18 ± 116.17**
2.2	52.18 ± 6.05	67.46 ± 7.31	145.06 ± 20.38	166.00 ± 22.03	225.68 ± 30.54	95.71 ± 21.21	244.41 ± 33.99	**313.10 ± 50.99**
2.3	59.10 ± 8.28	67.99 ± 10.03	180.42 ± 35.32	250.33 ± 32.45	312.19 ± 55.91	175.09 ± 39.24	483.25 ± 64.59	**495.56 ± 108.45**
2.4	62.42 ± 4.68	73.63 ± 5.48	195.23 ± 5.48	249.57 ± 31.06	256.32 ±37.12	147.52 ± 37.22	382.41 ± 64.92	**402.12 ± 119.72**
2.5	46.16 ± 7.05	59.61 ± 9.51	126.52 ± 25.52	136.87 ± 27.51	**236.22 ± 56.33**	66.14 ± 20.90	193.86 ± 43.01	232.75 ± 55.08
3.1	7.38 ± 5.47	10.03 ± 6.74	26.38 ± 20.27	22.17 ± 17.34	32.67 ± 22.58	32.90 ± 23.09	38.74 ± 27.82	**54.83 ± 40.71**
3.2	7.60 ± 2.48	9.16 ± 2.52	17.35 ± 5.99	17.76 ± 7.04	13.27 ± 3.45	18.89 ± 6.02	22.65 ± 6.69	**22.75 ± 6.45**
3.3	6.53 ± 4.63	7.83 ± 5.31	13.86 ± 10.11	16.37 ± 15.80	12.27 ± 9.95	16.37 ± 14.08	20.42 ± 18.42	**21.81 ± 20.28**
3.4	8.52 ± 3.18	10.38 ± 3.47	23.97 ± 10.25	23.91 ± 10.09	20.40 ± 7.94	23.25 ± 10.07	26.22 ±11.56	**30.52 ± 13.03**
3.5	5.37 ± 1.43	6.40 ± 1.56	12.88 ± 3.89	12.30 ± 3.80	11.14 ± 2.84	12.89 ± 3.78	14.52 ± 3.99	**15.86 ± 4.34**
3.6	5.10 ± 3.28	5.90 ± 3.82	10.89 ± 6.63	12.52 ± 8.34	9.23 ± 6.56	11.31 ± 7.38	12.43 ± 8.01	**13.26 ± 8.59**
3.7	6.56 ± 1.79	7.94 ± 1.96	13.47 ± 3.56	14.14 ± 4.82	13.40 ± 3.60	14.29 ± 3.78	16.41 ± 4.09	**18.00 ± 4.34**
3.8	7.83 ± 2.23	10.13 ± 2.49	25.58 ± 7.50	24.24 ± 8.60	23.46 ± 7.14	33.92 ± 8.22	39.62 ± 7.24	**54.74 ± 9.83**

Table S5 *SNR* improvement for the A2ASDOCT dataset. All the volumes were compounded using 4 consecutive frames. The results show the improvement with respect to the initial metrics presented in Table S1 (raw images). The best *SNR* in all the volumes are highlighted in bold. TNODE presented the best *SNR* improvement in all the volumes except Volumes 4.5 and 4.8 were PNLM had the best ratio. WCAN had the third best ratios.

Volume ID	MEAN	DNCNN	KSVD	WVMF	NAWT	PNLM	TNODE	WCAN
4.1	5.37	5.34	8.31	10.12	6.84	12.25	**12.54**	11.68
4.2	5.16	5.17	7.85	9.59	7.75	11.44	**11.70**	10.98
4.3	5.27	5.25	7.90	9.87	8.02	11.96	**11.98**	11.52
4.4	5.24	5.15	7.92	9.64	7.68	11.62	**11.80**	11.09
4.5	4.69	4.77	7.02	8.44	7.16	**9.77**	9.47	9.41
4.6	5.43	5.59	8.60	10.49	8.22	12.77	**13.08**	12.12
4.7	5.65	5.49	8.52	10.65	6.66	13.33	**13.48**	12.73
4.8	6.06	5.89	11.05	12.93	9.64	**21.62**	19.08	16.81
4.9	5.01	4.96	7.69	8.88	6.07	10.57	**10.60**	9.65
4.10	4.96	4.92	7.57	8.95	5.36	10.56	**10.62**	10.15
4.11	5.35	5.30	7.65	9.93	7.73	12.09	**12.14**	11.45
4.12	5.15	5.06	7.75	9.26	5.85	11.00	**11.19**	10.48
4.13	5.07	5.26	8.11	9.87	7.90	11.91	**12.14**	11.35
4.14	5.13	5.03	7.70	9.28	7.22	11.06	**11.28**	10.55
4.15	5.19	4.99	7.54	9.07	6.56	10.69	**10.99**	10.24
4.16	5.28	5.16	8.10	9.72	6.92	11.78	**11.99**	11.10
4.17	5.3	5.20	8.05	9.83	8.08	11.93	**12.21**	11.22

Table S6 *CNR* improvement for the A2ASDOCT dataset. All the volumes were compounded using 4 consecutive frames. The results show the improvement with respect to the initial metrics presented in Table S1 (raw images). The best *CNR* in all the volumes are highlighted in bold. WCAN presented the best *CNR* improvement in all the volumes except in Volume 4.9 where PNLM presented the best improvement.

Volume ID	MEAN	DNCNN	KSVD	WVMF	NAWT	PNLM	TNODE	WCAN
4.1	0.46	0.49	0.78	1.11	0.69	1.20	1.16	**1.24**
4.2	0.44	0.39	0.66	0.97	0.66	1.12	1.06	**1.15**
4.3	0.74	0.79	1.21	1.86	1.51	2.05	1.99	**2.21**
4.4	0.67	0.65	1.01	1.40	0.91	1.52	1.45	**1.54**
4.5	0.36	0.38	0.57	0.82	0.58	0.90	0.82	**0.92**
4.6	0.46	0.53	0.83	1.15	0.75	1.31	1.24	**1.32**
4.7	0.69	0.70	1.05	1.55	0.81	1.58	1.49	**1.71**
4.8	0.56	0.55	0.91	1.38	1.00	1.50	1.50	**1.63**
4.9	0.57	0.58	0.90	1.29	0.68	**1.42**	1.37	1.36
4.10	0.89	0.93	1.46	2.18	1.09	2.30	2.26	**2.46**
4.11	0.5	0.53	0.79	1.28	0.80	1.51	1.43	**1.53**
4.12	0.55	0.57	0.92	1.32	0.74	1.49	1.43	**1.50**
4.13	0.63	0.66	1.03	1.50	0.96	1.59	1.53	**1.66**
4.14	0.51	0.54	0.85	1.27	0.76	1.34	1.30	**1.39**
4.15	0.43	0.43	0.70	1.01	0.60	1.13	1.07	**1.18**
4.16	0.56	0.58	0.92	1.27	0.76	1.36	1.33	**1.39**
4.17	0.41	0.45	0.73	1.04	0.72	**1.22**	1.16	1.21

Table S7 *ENL* improvement for the A2ASDOCT dataset. All the volumes were compounded using 4 consecutive frames. The results show the improvement with respect to the initial metrics presented in Table S1 (raw images). The best *ENL* in all the volumes are highlighted in bold. WCAN presented the best ENL improvement in all the volumes except in Volumes 4.2, 4.5, 4.11, 4.12 and 4.14 where PNLM presented the best improvement and in Volume 4.9 where TNODE was first.

Volume ID	MEAN	DNCNN	KSVD	WVMF	NAWT	PNLM	TNODE	WCAN
4.1	22.02	22.62	47.39	73.00	40.81	90.41	97.15	**105.71**
4.2	25	25.01	65.16	119.23	57.23	**208.45**	203.91	203.82
4.3	32.75	34.50	65.28	140.44	101.89	145.49	152.06	**193.83**
4.4	26.33	25.73	52.03	89.15	48.32	92.63	95.00	**101.86**
4.5	22.16	22.20	55.86	110.13	57.08	**209.51**	188.61	195.13
4.6	23.69	24.49	56.12	93.62	46.95	113.46	114.66	**123.57**
4.7	27.93	28.02	50.58	91.39	46.19	80.09	78.94	**100.78**
4.8	20.6	20.78	38.00	75.86	52.69	78.23	80.94	**97.61**
4.9	16.54	16.57	34.12	64.30	23.01	84.28	**87.32**	81.92
4.10	34.75	37.12	70.50	164.07	56.01	145.72	158.06	**196.74**
4.11	18.31	18.31	38.41	91.80	40.32	**204.98**	192.90	184.91
4.12	30	30.02	75.49	135.46	63.13	**295.75**	276.12	219.84
4.13	27.63	28.98	57.57	104.82	56.19	107.19	110.42	**122.70**
4.14	24.93	25.47	56.38	108.29	48.99	**169.24**	158.31	153.88
4.15	27.22	26.97	68.18	134.56	50.04	174.33	172.65	**211.54**
4.16	23.98	24.37	49.01	75.37	37.07	79.81	84.95	**89.48**
4.17	24.40	24.95	62.03	106.27	55.33	**170.59**	162.52	169.55

Fig. S1 Results of the application of the WCAN method over frames from dataset MUW-1. Volumes 1.1–1.5 (see Table 2). using three B-scans at consecutive positions. (a) OCT raw image Volume 1.2 (b) WCAN image. (MP4, 8.3 MB).

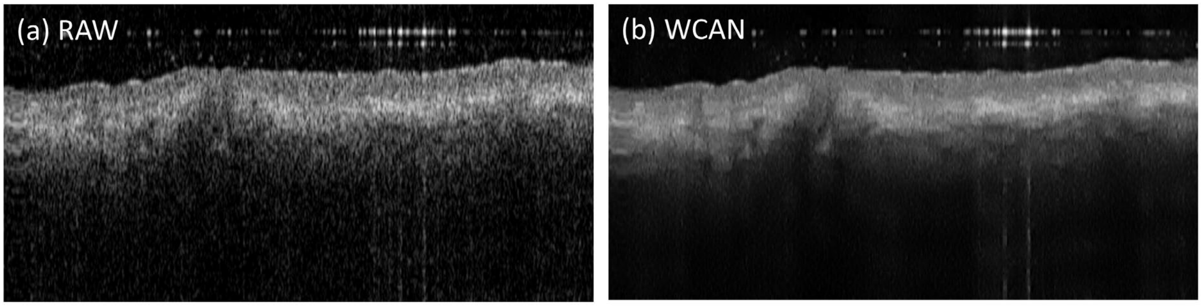

Fig. S2 Results of the application of the WCAN method over frames from dataset MUW-2, Volumes 2.1–2.5 (see Table 2), using three B-scans at consecutive positions. (a) OCT raw image Volume 2.5 (b) WCAN image. (MP4, 10.6 MB).

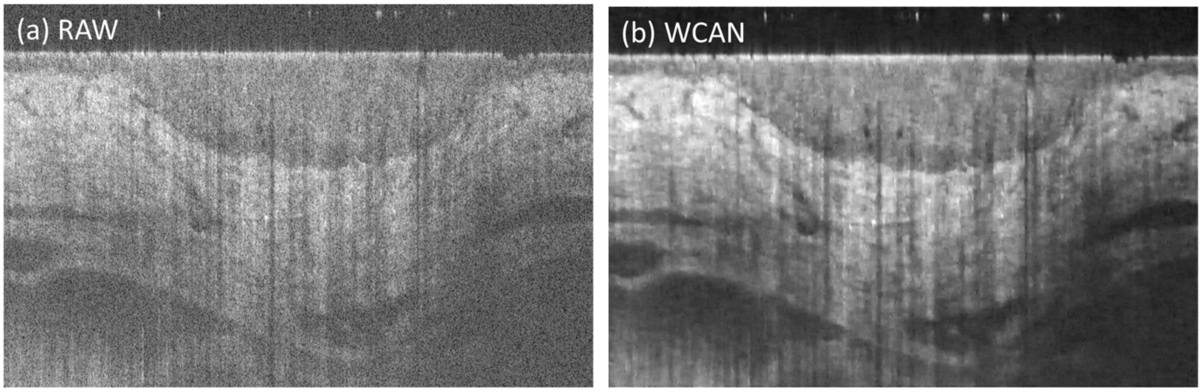

Fig. S3 Results of the application of the WCAN method over frames from dataset MUW-3, Volumes 3.1–3.5 (see Table 2), using two B-scans at consecutive positions. (a) OCT raw image Volume 3.3 (b) WCAN image. (MP4, 1.8 MB).

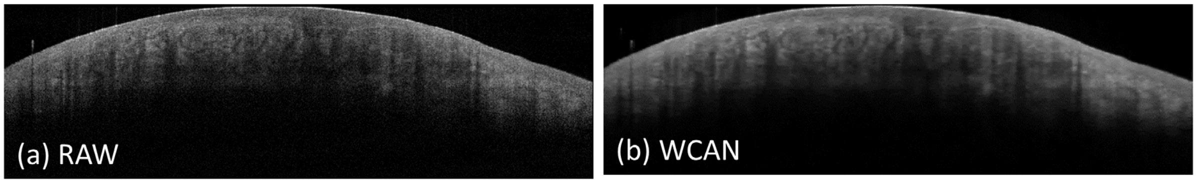

Fig. S4 Results of the application of the WCAN method over frames from dataset A2ASDOCT, Volumes 4.1–4.17 (see Table 2), using four B-scans at consecutive positions. (a) OCT raw image Volume 4.12 (b) WCAN image. (MP4, 9.9 MB).

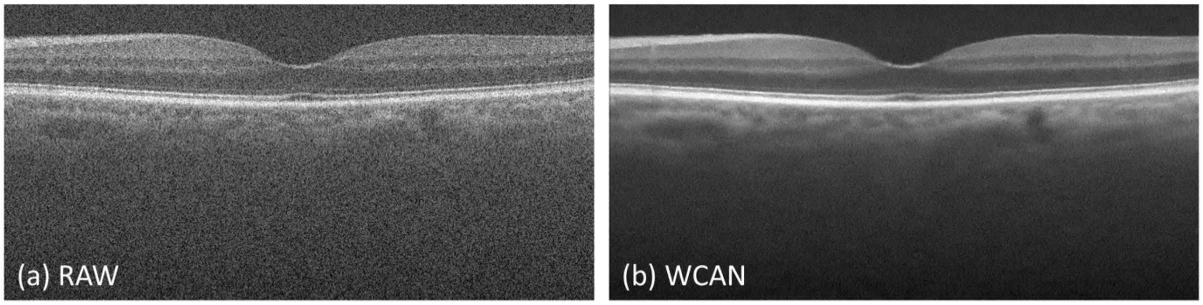

Supplement: Supplementary file 1 [file JBO_026_065001_SD001.pdf]
